# BFAST: An Alignment Tool for Large Scale Genome Resequencing

**DOI:** 10.1371/journal.pone.0007767

**Published:** 2009-11-11

**Authors:** Nils Homer, Barry Merriman, Stanley F. Nelson

**Affiliations:** 1 Department of Computer Science, University of California Los Angeles, Los Angeles, California, United States of America; 2 Department of Human Genetics, David Geffen School of Medicine, University of California Los Angeles, Los Angeles, California, United States of America; Baylor College of Medicine, United States of America

## Abstract

**Background:**

The new generation of massively parallel DNA sequencers, combined with the challenge of whole human genome resequencing, result in the need for rapid and accurate alignment of billions of short DNA sequence reads to a large reference genome. Speed is obviously of great importance, but equally important is maintaining alignment accuracy of short reads, in the 25–100 base range, in the presence of errors and true biological variation.

**Methodology:**

We introduce a new algorithm specifically optimized for this task, as well as a freely available implementation, BFAST, which can align data produced by any of current sequencing platforms, allows for user-customizable levels of speed and accuracy, supports paired end data, and provides for efficient parallel and multi-threaded computation on a computer cluster. The new method is based on creating flexible, efficient whole genome indexes to rapidly map reads to candidate alignment locations, with arbitrary multiple independent indexes allowed to achieve robustness against read errors and sequence variants. The final local alignment uses a Smith-Waterman method, with gaps to support the detection of small indels.

**Conclusions:**

We compare BFAST to a selection of large-scale alignment tools - BLAT, MAQ, SHRiMP, and SOAP - in terms of both speed and accuracy, using simulated and real-world datasets. We show BFAST can achieve substantially greater sensitivity of alignment in the context of errors and true variants, especially insertions and deletions, and minimize false mappings, while maintaining adequate speed compared to other current methods. We show BFAST can align the amount of data needed to fully resequence a human genome, one billion reads, with high sensitivity and accuracy, on a modest computer cluster in less than 24 hours. BFAST is available at http://bfast.sourceforge.net.

## Introduction

Recently developed massively parallel “next-generation” sequencing technologies have begun to replace the previously dominant Sanger sequencing technology [1]–[3] for large-scale sequencing projects. Technologies like Illumina's Genome Analyzer [4], Roche's 454 [5], [6], and ABI's SOLiD [6] are able to generate billions of bases of total sequence in a matter of days. These technologies generate relatively short reads, typically from a few tens to a few hundred bases in length, with a general inverse relation between the total number of reads and the read length. In the context of whole human genome resequencing, on the order of a billion short reads are required to accurately resequence an individual genome, and this creates an unprecedented alignment problem of aligning this many reads to the reference human genome on a practical timescale of days. Using established dynamic programming algorithms [7] to align reads to the entire human genome is grossly impractical, since the computational cost is proportional to the target size. To reduce the cost resulting from a large alignment target, many algorithms have been developed that rapidly reduce the size of the search target for aligning a given read. This is typically performed by passing it through an index of the reference genome [8]–[14], or by indexing the reads and searching the reference genome [15], [16]. Using an indexing approach, all the algorithms reduce the time complexity by trading off accuracy and completeness of the search for candidate alignment locations (CALs) to which local alignment is performed. In aggregate, these algorithms are either limited in performance time or accuracy, and can lead to the inability to detect the biologically relevant variants (predominantly single base mismatches, and insertions or deletions of 3 bases or greater), and with some algorithms imposing a limit on the length of the read that can be considered (see Supplemental Materials S1).

To address the general speed and accuracy limitations, especially in the context of short reads with potential errors, single base variants and insertion/deletions, of the currently described alignment tools, we developed a new algorithm and associated software tool called BFAST (for BLAT-like Fast Accurate Search Tool). BFAST can align giga-scale short read sets with comparable or better speed compared to existing methods, while maintaining higher sensitivity and accuracy for deletions/insertions. While our algorithm is meant to facilitate practical human whole-genome resequencing, it is not restricted to this task and should prove useful for many problems that involve aligning massive numbers of short reads to a reference genome. Here we describe the basic approach and demonstrate BFAST robustness to detect an insertion or deletion, relative to other methods in the real world context of sequence errors and polymorphisms. BFAST also has the ability to be customized for given time or sensitivity requirements, since we are able to *a priori* measure the theoretical sensitivity against mismatches and variants before performing alignment based on the underlying choice of genomic indexes.

## Results

### General Design of BFAST

BFAST is a powerful and complete means to perform billions of short sequence alignments within the context of large genomes in a highly sensitive and tunable manner. BFAST performs alignment in two steps. First, using multiple indexes of the reference genome, BFAST identifies candidate alignment locations (CALs) for each read. Next, the reads at each CAL are further aligned using gapped local alignment to identify the best match. These processes are supported for direct sequence reads (the typical output of platforms based on sequencing-by-synthesis, such as the Illumina, 454 or Helico sequencers) as well as reads in two base color encoded form, which is the primary output of the ligation-based ABI SOLiD platform. The first step can be substantially tuned for various levels of sensitivity, accuracy, and speed based on the users goals and the error properties of the data. The second step allows for full completion of alignment - i.e. considers all possibilities including SNPs, insertions, deletions, as well as color errors (the latter is relevant for ABI SOLiD data) [7], [17]. Gapped local alignment is considerably more computationally expensive than ungapped alignment, especially for ABI SOLiD color space data, but improves our ability to find SNPs, insertions, deletions, and compensate for color errors simultaneously. In addition, allowing for fully gapped local alignment makes BFAST suitable for data that contains indels as a common error mode (for example, in the 454 or Helicos platforms). Additionally, these indexes are expensive to store in main memory (RAM), and therefore should be fully utilized by generating as many lookups per read as possible, providing sensitivity to errors and variants. What makes BFAST unique is that prior methods neglect one of followings areas that are inherent and key to the highly sensitive and relatively fast lookup process enabled within the design principles of BFAST: 1) use of multiple indexes, 2) full use of indexes when loaded into RAM, or 3) perform fully gapped local alignment (see Supplemental Materials S1).

**Figure 1:**
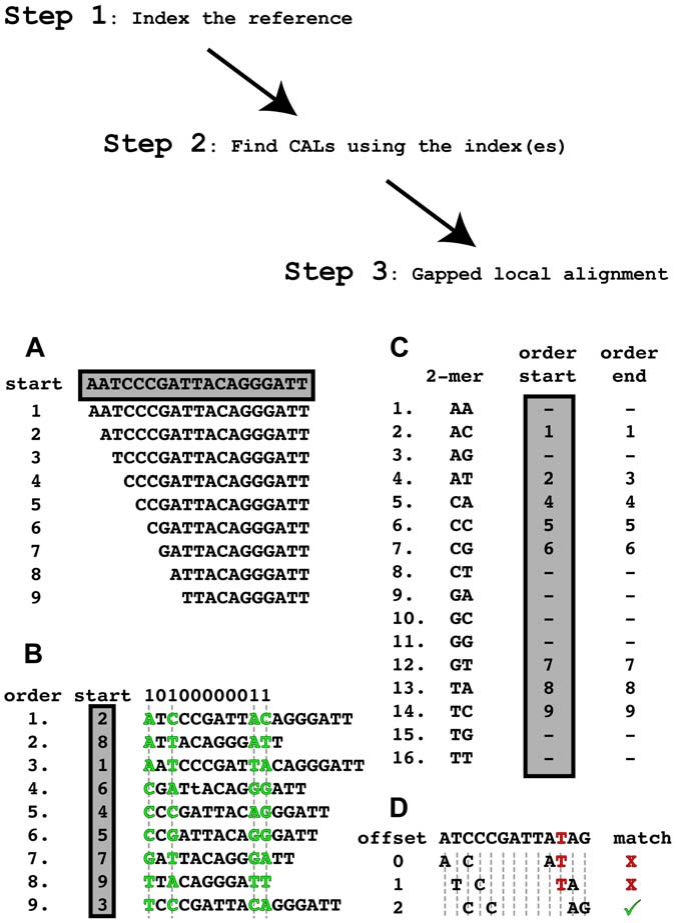
Algorithmic steps and underlying data structure used by BFAST. BFAST has three sequential steps: create indexes of the reference, find CALs (candidate alignment locations) using the indexes, and perform gapped local alignment. Gapped local alignment is performed on all possible CALs in order to identify the best possible alignment. Thus, a highly sensitive and comprehensive search step is followed by an integrated local alignment to maintain high sensitivity and high accuracy. The indexes can be reused on new sets of reads when the reference remains the same. In Panels **A–D** we detail the underlying data structure and storage format used to represent and access an index. **Panel A** represents an example genome (shaded region) to be indexed and a list of all suffixes of the genome with length greater than 10. The shaded regions are the only portions that must actually be stored in RAM: the genome sequence, the ordering of the suffix list, and the entries of the *k*-mer hash lookup. **Panel B** shows a mask with a key-size of 4 and width 11 to be indexed (10100000011), with a list of all suffixes ordered by the 4-mer keys defined by the mask that are possible from the target sequence. The vertical shaded region in **Panel B** gives the original ordering of the suffixes, or alternatively their start positions in the genome. A 2-mer hash table is shown in **Panel C** and is used to rapidly lookup 4-mer entries in the index with the key based on the 2-mer prefix of the 4-mer word. This hash, or an index into the index, returns a range (order start and order end) over the ordered suffix list in **Panel B**. **Panel D** shows the possible lookups for a 13-base read using the index defined in **Panels A, B, and C**. There is an error in the seventh position shown in red. Using the index, three different 4-mers can be defined by sliding the mask along the read. To lookup a given 4-mer, such as ATAG, the hash table is first consulted to find that the prefix AT extends from entry 2 to entry 3 in the index. Since this is not a unique range (i.e. the start and end are equal), the index is bisected using the range from the hash lookup, until all positions with ATAG are located. The read error (or mutation) interferes with alignment in that only one of the three 4-mers are found in this created index, but still yields the proper location for this read in the genome, further indexes would make reads with more errors/mutations mappable correctly. In practice longer keys are used.

**Figure 2:**
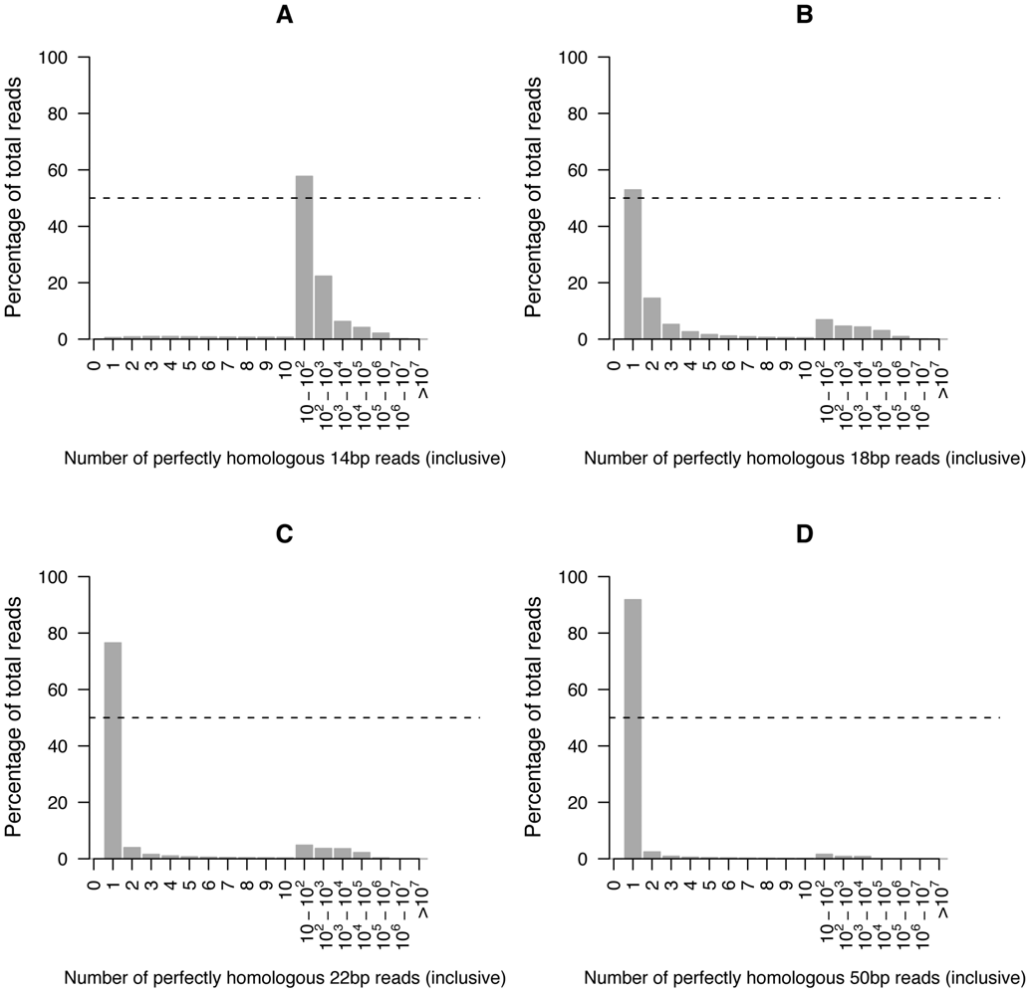
Distribution of short sequence CALs to the human genome at various *k*-mer keys. For varying key sizes, *k*, the number of lookup locations for each *k*-mer key from the Human reference genome was calculated, forward and reverse complementary sequence included (∼6·10^9^ keys). The figures show the computed percentage of keys that have a given number of genomic locations, the equivalent of CALs for index lookup.

The novel contribution of BFAST is the CAL search step, where we tabulate a list of CALs for each read with the goal to include the true (or correct) location within the CALs. BFAST uses multiple indexes of the reference to increase sensitivity of alignment. Each index is a space-efficient suffix array of the reference genome (see Figure 1). An index is defined by a spaced seed (or mask), a string of 0s and 1s that start and end with a 1, that define the bases in the read considered during the lookup in the index (see Table 1 for a complete example optimized for the human genome). We refer to the number of ones in the mask as the key size (*k*), and the total number of ones and zeros as the key width (*w*). The number of CALs returned depends on the key size as well as the complexity and repetiveness of the target genome, with longer key sizes making the lookup more unique (on average) but generating fewer lookups resulting in reduced sensitivity (Figure 2). The number of matching CALs from the global search for various length *k*-mer keys from the Human reference genome, including the forward and reverse complementary sequence (∼6·10^9^ keys), indicates that for the non-repetitive part of the genome, a key size of *k* = 18–22 is sufficient for obtaining a unique lookup the majority of the time, but that a key size of 14 or lower would rarely return a unique CAL (Figure 2). Conversely, a key size of 50 would primarily return a single CAL. Since we are able to apply a mask at every offset from the start of the read, we try to minimize the key size (and key width) to generate more lookups. Therefore, we choose a large enough key size such that the lookup is unique but small enough such that multiple lookups can be produced. The repetitive portion of the genome has a large number of CALs as expected and indicated at the tail populations in the histograms with 10^2^–10^7^ locations (i.e. *Alu* elements). Therefore, for the human genome we prefer *k* = 18 for short reads (40 bp<) and *k* = 22 for longer reads (≤40 bp) to balance sensitivity and the uniqueness of the lookup. Shorter keys are useful for shorter reads as the number of offsets possible is greater and improves sensitivity. BFAST implements a hash into the index to reduce the lookup time (see Figure 1) that consists of indexing the first *j* bases (the hash width) of the reference indexes with *j*≤*k*. The hash width is always shorter than the key size.

**Table 1:** Index sets used by the four modes of BFAST for 50 base pair reads from the human genome.

Mask	Key size (k) and key width (w)	1	2	3	4
M_1_ = 111111111111111111	(k = 22, w = 22)	M	M	M	M
M_2_ = 1111101110111010100101011011111	(k = 22, w = 31)	M	S		
M_3_ = 1011110101101001011000011010001111111	(k = 22, w = 37)	M	S		
M_4_ = 10111001101001100100111101010001011111	(k = 22, w = 38)	M	S		
M_5_ = 11111011011101111011111111	(k = 22, w = 26)	M			
M_6_ = 111111100101001000101111101110111	(k = 22, w = 33)	M			
M_7_ = 11110101110010100010101101010111111	(k = 22, w = 35)	M			
M_8_ = 111101101011011001100000101101001011101	(k = 22, w = 39)	M			
M_9_ = 1111011010001000110101100101100110100111	(k = 22, w = 40)	M			
M_10_ = 1111010010110110101110010110111011	(k = 22, w = 34)	M			

The masks sets for each of the four BFAST modes: *accurate*, *moderate accuracy*, *moderate speed*, and *fast* are listed under the columns 1, 2, 3, and 4 respectively optimized for 50 base pair reads from the human genome. We prefer to use the *accurate* setting when aligning to the human genome. The masks used in the main indexes are listed as M, and the masks used in the secondary indexes are listed as S. The key-sizes and key-widths are indicated next to each mask.

For the human genome and 50 bp reads, we typically use ten indexes with key size 22 with variable key widths, and a hash width of 14. The ten masks for the indexes are chosen using a simple greedy algorithm to maximize sensitivity gain from each additional mask (see Supplemental Materials S1 for more details). Other methods to find optimal spaced-seeds are available[18]–[20]. For 50 bp Illumina data from a human genome, ten indexes and their associated masks were optimized for sensitivity (Table 1). The RAM required to load each index for the human genome serially is approximately 17Gb, and the practical total computer RAM requirement for optimal performance is 24Gb, which is readily feasible with current computer hardware. BFAST also has the ability to split an index into 4^n^ pieces (this number is chosen for efficiency), although the lookup phase of the alignment takes somewhat longer because each read will undergo a lookup attempt for each partial index. In this mode, BFAST for the human genome can run well on computers with as little as 4Gb of RAM. Since the key width of an index (*w*) here is less than the read length, we are able to apply the mask at all possible starting positions offset from the beginning of the read to increase the number of lookups performed per read and thus further improve sensitivity. Additionally, to reduce the running time of BFAST at a small reduction in sensitivity, we impose two upper limits during the search. The first requires that a single lookup be ignored if it returns more than *K* CALs (we use *K* = 8 for the human genome). The second requires that a read be ignored if the list of CALs for a read grows beyond length *M* (*M = *1280 for the ten indexes with the human genome presented here). The number of CALs can be reduced, which improves performance time but decreases sensitivity. Further discussion of appropriate settings of *K* and *M* can be found in the Supplemental Materials S1 (see Figure S4). Within BFAST, paired-end or mate-pair data are aligned independently for each end while retaining the paired-end or mate-pair annotation, which provides flexibility in downstream analyses and permits us to assess the performance of the short read alignment.

In the Supplemental Materials S1, we examine the design choices leading to conception of BFAST, including its basis on BLAT[10]. We also detail the motivation for the parameters mentioned above when aligning to the human genome reference. Finally, in the Supplemental Materials S1, we examine the ability of BFAST to be tuned trading off time for sensitivity. In practice we prefer sensitivity to speed, since it is our goal for whole-genome human resequencing to discover variants in the presence of errors.

### Performance Results

In order to assess the performance of BFAST, we compare BFAST to other available software for the ability to correctly map short reads in the context of different numbers and types of base differences from the consensus human genome such that the use of BFAST for variant discovery in the human genome is apparent and can be reasonably inferred. For this, both simulated and real world datasets are used. In all comparisons, we evaluate the ability of a method to sensitively and accurately find the correct candidate alignment location (CAL) for a given read during the global alignment step. Within BFAST, the CAL is followed by a gapped Smith-Waterman local alignment for each CAL returned, and subsequent assessment of the best alignment to the consensus genome of all possible CALs. In this approach, BFAST sacrifices some speed in the CAL identification process in exchange for completeness and results in gapped local alignment being performed on all CALs. Most other methods do not perform gapped local alignment (bowtie, and SOAP), or only in exceptional circumstances with paired end data (MAQ). These methods sacrifice completeness for speed, which may be desirable under some scenarios, but will also lessen the ability to identify true variants. We note that in our simulations we do not require that the errors, SNPs, or indels be identified correctly or even found by other methods, but instead require only that the read is placed within 10 bases of the true location. Thus we are only evaluating the methods on their ability to find a correct CAL during the genome-wide global alignment, and not their ability to finish this with correct variant detection. We take this approach for two reasons: first, unlike BFAST, many of the aligners do not perform the full gapped alignment that would be necessary to properly identify indels, and yet we still wish to compare their performance properties to the extent possible. Second, in the context of a resequencing project, variant detection is ultimately a process in which alignment is only the first phase, and which could employ diverse analytical methodologies, such as local reassembly, to return final variant calls. Thus it is most critical to properly position the reads, via a correct CAL, but it is not fundamentally critical for the aligner to fully detect the variant within the read, although it certainly is a useful capacity and obviously can help in finding the approximate location. We also note as well that it is not our intent here to perform a comprehensive comparison of aligners, or to exhaustively compare all available methods to the many possible modes (index choices) of BFAST, but instead to use a representative sample of other reported methods to put the performance of BFAST in context, and to illustrate the key areas where BFAST provides a compelling advantage in the search for variants in the context of the human genome.

**Figure 3:**
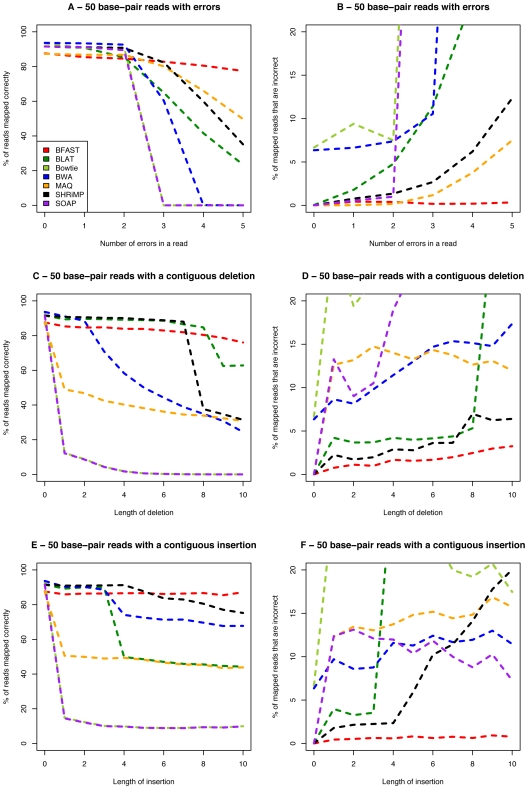
Evaluation of alignment algorithms from simulated 50 base pair reads. 10,000 50 bp reads each fitting to different read classes were aligned using indicated algorithms back to the human genome. Sensitivity is defined as the percent of all reads that were mapped correctly and is plotted on the y-axis in panels A, C, and E. The percentage of reads that are mapped to an incorrect location are plotted on the y axis in panels B, D, and F. The x-axis of panels A and B maps the number of sequence differences in the reads relative to the consensus genome. The x-axis plots the length of a contiguous deletion in panels C and D, and the length of a contiguous insertion in panels E and F. The alignment method is indicated as colored lines per the key within the first figure panel.

BFAST was compared under various models of errors and insertion/deletions to other alignment tools to determine if there were observable improvements in alignment in the simulated datasets (Figure 3). We evaluated BFAST, along with BLAT [10], bowtie[13], BWA[14], MAQ[16], SHRiMP[15], and SOAP[11], using various simulated datasets in order to demonstrate practical and beneficial features that are unique to BFAST. We used the same settings for these algorithms across the evaluations. Each method handles reads that map to different locations differently. For instance, reads with are equally mapped to multiple locations in MAQ are given a quality mapping score of zero and are here considered ‘unmapped’. In BFAST, up to 384 CALs are considered and are sorted out by gapped local alignment. If a read maps best to a given location relative to the other CALs, it is considered ‘mapped’ and may thus inflate the error rate if indeed mismapped. If a read is equally mapped to two alternative locations in the genome for BFAST after local alignment it is considered ‘unmapped’. This permits a more direct comparison of the global search in the context of false mapping rates. To evaluate the sensitivity and accuracy of the various alignment algorithms, we simulated from the human genome various 50 base-pair read classes. Each read class contained reads with a fixed number of introduced random errors relative to the consensus genome sequence. Both single base differences (dominantly from errors of the machines) and various length insertions or deletions were considered separately or together in order to obtain accuracy statistics to compare with theoretical accuracy (Figure 3). Further, since the distribution of the simulated read classes is not necessarily representative of real data from massively parallel sequencers, we also evaluated the performance and running times of alignment algorithms on four real-world datasets from two different sequencing platforms: Illumina and ABI SOLiD (see Supplemental Materials S1 and Table 2) generated within the UCLA DNA Microarray Facility. In these comparisons, BLAT, bowtie, and SOAP are not possible to include for the ABI SOLiD data since these software do not support alignment in color space. A simple implementation of a mapping quality filter is implemented when possible to remove low quality mappings that could artificially inflate sensitivity (see Supplemental Materials S1). More stringent post-alignment filters can be used to reduce the false-mapping rates of all methods further, but will result in some decrease in sensitivity. Further, some methods have more sophisticated post-alignment filters, which are not considered here. For the most part these additional filters will remove aligned data lessening sensitivity but improving accuracy. Because both sensitivity and false mapping rates are calculated on the datasets, the methods can be directly compared in terms of the fraction of reads mapped at a given error rate.

**Table 2:** Timing results of alignment algorithms on four different real-world datasets.

	Illumina 10.9 M 36 bp reads	Illumina 10.9 M 36 bp reads	Illumina 3.5 M 55 bp reads	Illumina 3.5 M 55 bp reads	ABI SOLiD 1 M 25 bp read	ABI SOLiD 1 M 25 bp read	ABI SOLiD 1 M 50 bp read	ABI SOLiD 1 M 50 bp read
	Time (s)	% mapped	Time (s)	% mapped	Time (s)	% mapped	Time (s)	% mapped
**BFAST**	43,775	32.1	47,474	69.6	9,590	66	42,856	72.5
BLAT*	68,758	24.3	6,735,069	77.4	NA	NA	NA	NA
**Bowtie**	2,270	13.1	857	55.7	NA	NA	NA	NA
**BWA**	7,682	16	4,883	59.3	21,179	74.7	845	47.8
**MAQ**	8,607	28.7	126,541	73.6	7,602	63.6	6,680	68.1
SHRiMP*	186,764	14.9	324,380	83.3	2,977	2.4	32,644	70.4
**SOAP**	11,938	13.3	131,248	62.4	NA	NA	NA	NA

For four different real-world datasets sequenced on an Illumina GA1 sequencer, Illumina GAII and an ABI SOLiD sequencer, the run time and the fraction of reads mapped were tallied. Settings for each method are detailed in methods. We extrapolated these values for those methods denoted with an asterisk (*) (see Supplemental Materials S1).

We first consider the relative sensitivity and accuracy of read mapping with variable numbers of mismatches to consensus (Figure 3A and 3B). The ability to map reads with no errors is relatively trivial and differs between methods due mainly to the upper limit on the number CALs generated before a read is ignored as well as the stringency of post-alignment filters. As expected from the design of the algorithm, BFAST has substantially better sensitivity than the other methods when considering combinations of single base errors. The relative improvement in sensitivity of BFAST is demonstrated for the mapping of 50 bp reads with 3 or more mismatches to the consensus genome. For instance, BFAST is able to align 50 mer reads with 5 errors to the human genome with 80% sensitivity and under 1% mismapping rate without the use of paired end data far exceeding other implemented mapping algorithms. The dominant error mode for the most massively parallel sequencers is single base miscalls. Thus, BFAST permits a larger fraction of reads with errors to be correctly aligned. Further, in some biological contexts, even in the absence of machine errors, BFAST would improve correct variant detection (such as highly polymorphic locations of the human genome like at the HLA locus).

To further highlight particular areas of performance improvement sought in the design of BFAST, we aligned 50-mer reads with randomly inserted variable lengths of deletions (Figure 3C, 3D) and insertions (Figure 3E, 3F). With up to 10 base deletions up to 80% of the reads are accurately placed by BFAST with under at 3% mismapping rate. The mismapping rate and sensitivity with 10 base deletions is better than the other methods. With up to 10 base insertions, about 90% of the reads are correctly placed with BFAST with under a 1% mismapping rate. Again under these conditions, BFAST is superior to other mapping tools. Each method could be further tuned to improve aspects of the alignment process, but we note that under the single conditions shown here that BFAST has equivalent or better sensitivity and mismapping rates relative to other methods over a range of single base differences and indel sizes. Thus, in the practical implementation for variant calling a single implementation of BFAST suffices to identify virtually all variant classes and be robust against machine read errors. Both BLAT and SHRiMP were able to sensitively map small insertions and deletions in 50 bp reads as accurately as BFAST, but dropped in sensitivity after the insertion or deletion reached a given length (Figure 3C and 3E). BWA's sensitivity was comparable to BFAST when mapping up to a 2 bp deletion but declined gradually afterwards. Furthermore, BWA was able to sensitively align a 3 bp insertion comparable to BFAST, but suffered on average a 20% decrease in power compared to BFAST on insertions longer than 3 bp with a higher mismapping rate. Both Bowtie and SOAP, due to their ungapped alignment processes, were able to map only a negligible amount of reads with an insertion or deletion. It is interesting to note that MAQ typically finds indels by performing gapped local alignment on paired end data. This requires that one end is mapped with confidence and the other end is not mapped with confidence but with the latter end instead placed within a specified distance away from the former end corresponding to the expected insert size distribution. This is a reasonable approach when paired end data are available and we wish to trade off completeness for speed, but does result in decreased ability to detect insertions and deletions. This could be mediated by always performing gapped local alignment similar to that implemented within BFAST. Further, performing multiple iterative rounds of global and local alignment, and perhaps even local reassembly, will benefit the overall alignment sensitivity and accuracy of all methods; however, this is not evaluated here. We also observed for ABI SOLiD color space data that BWA does not find longer insertions or deletions as it does for Illumina data (results not shown), which limits it usefulness when searching for indels on the ABI SOLiD platform.

**Figure 4:**
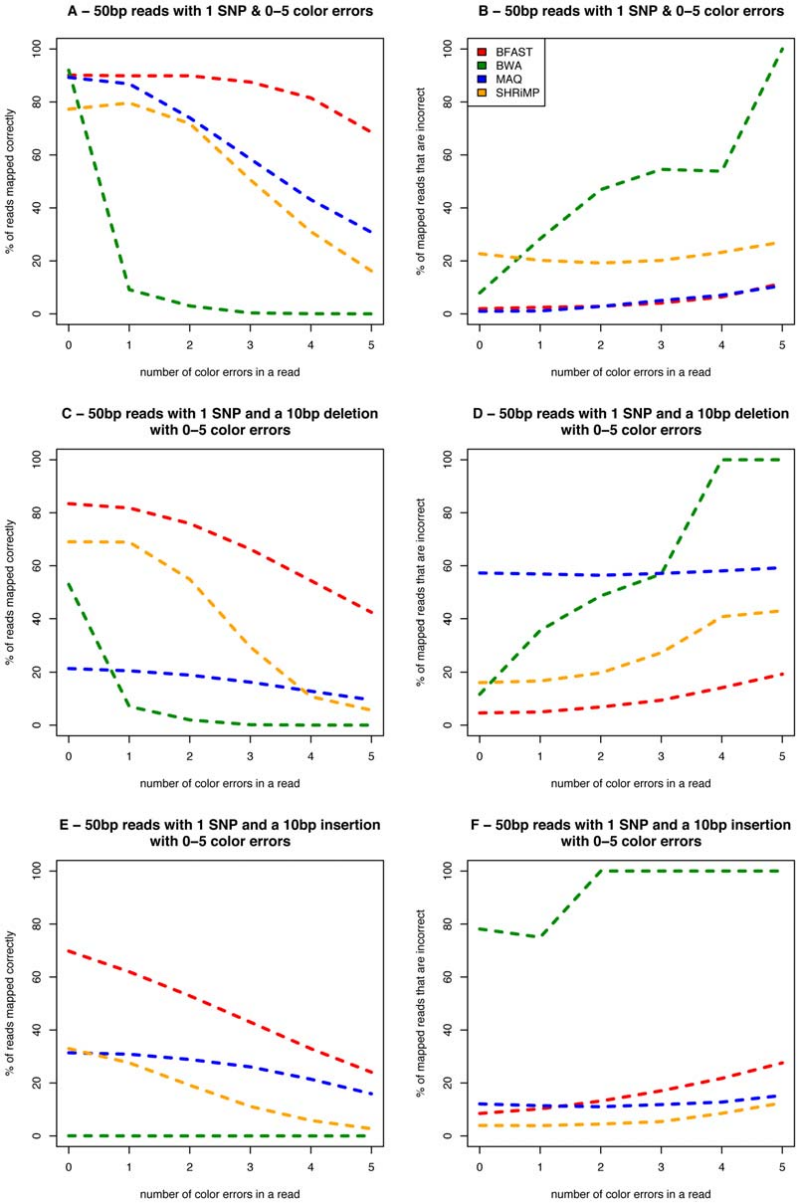
Evaluation of alignment algorithms from simulated 50 base pair color space reads. 10,000 50 base color space reads were simulated from the human genome in different read classes to assess sensitivity and accuracy of BFAST. Sensitivity is defined as the percent of all reads that were mapped correctly and is plotted on the y axis in panels A, C, and E. The percentage of reads that are mapped to an incorrect location are plotted on the y axis in panels B, D, and F. The X axis of all panels plots the number of color differences in the reads relative to the consensus genome for a variety of models. Panels A and B plot mapping of reads with a single SNP in addition to the color errors. Panels C and D plot mapping of reads with a single 10 bp deletion in addition to the color errors and single SNP. Panels E and F plot mapping of reads with a single 10 bp insertion in addition to the color errors and single SNP. The alignment method is indicated as colored lines per the key within the first figure panel.

BFAST and some alignment tools have been implemented to align two base encoded reads generated on the ABI SOLiD platform. Since both BWA and MAQ use color quality scores to aid in local alignment, we give simulated color errors a color quality of 20, and 30 otherwise. The mapping of color space reads were evaluated in a different manner as a single true base difference from the genome results in two color errors and a typical machine error results in a single color error. We thus create random read sets with a single SNP and 0–5 color errors (Figure 4A,4B) to demonstrate the ability of BFAST to identify the correct location even in the context of multiple machine errors. Over 80% of reads with up to 4 color errors and a SNP are correctly mapped with a mismapping rate of under 5%. At higher error rates BFAST identified the correct location for twice as many reads as the closest other algorithm implemented in MAQ. All methods performed well with no errors. For comparisons sake a read with 2 SNPs and one color space errors would be equivalent to 4 color errors in Figure 4A. Thus, BFAST sensitively aligns color space data even in the context of multiple variants with a low false mapping rate.

Ten base insertions and deletions are most sensitively detected by BFAST as well as demonstrated in figure 4C–F under a variety of color space errors. In the case of three color errors (the equivalent of 6% error), BFAST is able to align 87.5% of reads with one SNP, 66.2% of reads with one SNP and a 10 bp deletion, and 43.0% of reads with one SNP and a 10 bp insertion. Under settings that permit the higher fraction of correct placement of reads with BFAST, the false mapping rate is also lower than other methods, with the exception being the case of insertions, where SHRiMP has a lower false mapping rate (with much decreased sensitivity). While more stringent mapping quality filters can reduce these false-mapping rates, these filters would reduce the sensitivity of each of the other methods.

In Table 2 we evaluated the performance or running times of these algorithms on four real-world datasets from two different sequencing platforms: 10.9 million 36 bp reads from an Illumina 1G sequencer, 3.5 million 55 bp reads from an Illumina GAII sequence, 1 million 25 bp reads from an ABI SOLiD Sequencer, and 1 million 50 bp reads from an ABI SOLiD sequencer. The first Illumina dataset sequenced various PCR fragments from the human genome, the second Illumina dataset sequenced random fragments from a normal human genome, and the two ABI Solid datasets sequenced human genomic DNA.

Based largely on the more comprehensive look up strategies and implementation of gapped local alignment, BFAST has one of the slowest running times compared to other methods. This is mainly due to the specific settings used to maximize sensitivity to variants and errors within BFAST, as well as the time spent loading the 10 genomic indexes, which is included in the running time of BFAST. For larger scale datasets such as a whole-genome shotgun resequencing, we are able to partition the billions of reads such that the search and align times dominate relative to the loading times. Thus, BFAST does not scale linearly with these reported times. Although the running times of BFAST are slower in the majority of cases compared to other methods, when considering the need for sensitivity and accuracy of the alignment process in the context of whole-genome resequencing (see simulations above), BFAST has attractive advantages including the ability to be implemented in a parallel computational environment.

## Discussion

We have created a new sequence alignment tool that is specifically designed to meet the challenges of practical whole human genome resequencing using short read data (25–100 bases). BFAST has been implemented completely for the handling of large datasets from the ABI SOLiD color space reads as well as direct sequence reads possible with other platforms. The estimated scale of the problem–aligning a billion reads per day on a moderate computer cluster–obviously demands extremely fast alignment algorithms, but more importantly demands high accuracy of the alignments (i.e. obtaining the true alignment of a read, among the many various candidates). This is fundamental because the purpose of resequencing is to locate the variants, including insertions and deletions, relative to a reference genome, and to distinguish real genomic variants from inevitable sequencing errors. Thus, it is critical to find the true alignments of reads that do not match perfectly to the reference, so that sequencing errors can be filtered out via multiple coverage, and so that real variants will not go undetected due to systematic under-mapping of reads with the variant. In practice, then, a suitable method must be accurate to meet this challenge and still perform its operations under realistic computer hardware configurations and in reasonable time, and this was the underlying design goal of BFAST.

At a practical level, the BFAST performance tests presented here demonstrate various modes of accuracy of alignment for both simulated data and real-world datasets from both the Illumina and ABI SOLiD sequencing platforms. We demonstrate that BFAST is able to substantially increase the number of reads correctly placed in the genome when containing short insertions and deletions and various numbers of errors while maintaining a lower false alignment rate than any other alignment tools. We are able to map sensitively reads that have up to 10% of the bases in the reads being errors, as well as, reads that contain insertions and deletions up to 10 base pairs with modest error rates. This is critical for identifying indels within the genome of interest, and for optimizing independent alignments of paired end reads to identify structural variants. We demonstrate that BFAST is relatively robust to a wide range of errors. Under key conditions, BFAST is improves the likelihood of correct genomic placement of individual reads over a wide variety of scenarios.

While BFAST is highly sensitive with the use of 10 indexes, as shown here, if the sensitivity were not satisfactory for a given purpose, to the addition of additional genomic indexes would improve the sensitivity with a modest increase in the computational time. This relative error or variant tolerance of BFAST is highly relevant to two scenarios. First, efforts are being extended to sequence new genomes of species for which a closely related species genomic sequence currently exists. The alignment of the generated short read data must be aligned accurately to use the prior genome scaffold in the presence of unknown base differences. Second, the implementation of new and perhaps more error prone sequencing methods that may generate even larger amounts of data than current methods but with a higher error rate. BFAST will be able to more effectively align these poorer quality reads and render new technologies more useful.

The comparison of BFAST to other methods here–MAQ, BWA, Bowtie, SHRiMP, SOAP and BLAT–is not meant to be an exhaustive comparison to all other available programs, nor is it meant to imply that these particular methods cannot provide satisfactory results for large scale resequencing alignment problems. Rather, it is simply to provide a practical context for judging the speed and accuracy of BFAST demonstrate that any method used to align short reads should be assessed for its sensitivity and accuracy in the presence of multiple variants. The examples shown here illustrate that there can be a substantial decreases in accuracy of mapping for certain types of variants, such as small indels, or higher numbers of mismatches, which may be biologically important. Given the complexity of the possible settings, these methods comparison are always incomplete. For instance, we do not evaluate scenarios that would allow the evaluation of certain heuristic choices of the other methods, deferring to the design principle of completeness as motivation for this omission. We note that for many biologically relevant mutation classes that the accuracy of placement by BFAST with 10 genomic indexes is 80–90% thus there is little to be gained by iterative or heuristic processes.

Because BFAST uses multiple gapped indexes, BFAST is able to sensitively identify potential alignment positions. Only reads with under 384 CALs are subsequently evaluated by the powerful gapped local alignment algorithm, which distinguishes reads with identical mapping to multiple locations versus reads with a best matched location. By enforcing an upper limit on the number of CALs, BFAST could potentially ignore reads that map to multiple locations but could be placed within the human genome. Thus, in the interests of higher sensitivity alignments, a key area of potential improvement would be to improve the CAL search step with specialized indexes, such that the correct location is within a more limited set of CALs relative to the current implementation of BFAST.

Any method that underperforms in sensitivity compared to BFAST would need to reduce the false mapping rate to show improvement over BFAST beyond performance. This new method would need to improve the CAL search step, as the gapped local alignment is optimal, such that for some read the new method includes the correct location in its set of CALs when BFAST does not. This correct location would then be identified by the powerful gapped local alignment. This requires the new method to have greater sensitivity, when the opposite was assumed. As BFAST is tunable for any level of sensitivity, we are able to sensitively map reads as well as controlling for false positives.

The choice of alignment tool for each biologist should be tailored to the goals of the sequencing project. While under the specified parameters, the other methods can be somewhat faster for some datasets and slower for others; the fraction of data mapped was typically less than that achieved using BFAST. We note that BFAST does not have default speed or accuracy parameters per se but rather allows the design of index sets with a broad range of speed/accuracy trade offs. The software distribution includes an extensive family of pre-optimized index set masks from which subsets can be selected given any desired accuracy, as specified in the Supplemental Materials S1 (Table S3–S6). Our experience with aligning short read data (25–100 base-pair reads) has revealed that a major problem is that level of accuracy actually being achieved is not known in advance, i.e. what variants will be missed or underrepresented by the alignment process. We stress the need to assess accuracy for short read alignment as part of any such resequencing effort. If the alignment method is not able to sensitively align, then some categories of mutations will be completely missed.

We offer the BFAST program as a new tool in the available for processing massively parallel sequence data. The BFAST program is freely available at http://bfast.sourceforge.net, and is fully intended for use in production-level whole human genome resequencing efforts, such as are now underway in our own lab. For this reason, we have taken care with the implementation to make it portable, documented, independent of the sequencing platform, and targeted to run efficiently on a cluster. In practice, BFAST can align 1 billion Illumina 55 base-pair reads in 24 hour period on a cluster containing 156 computer computational cores, with suitable amounts of RAM, which makes these alignments practical from a computer hardware point of view. In particular, 20-node cluster with each node consisting of a dual quad core CPU with 24Gb RAM would suffice. We note that the benchmarks reported here ignored multi-threaded computation, and parallel computation, both of which BFAST supports, which in practice proportionately reduces the number of independent nodes required, when multi-CPU nodes and multi-core CPUs are available, as is widely the case. Further, BFAST is implemented in such a fashion that large-scale alignments can be successfully performed in computation clusters where only a fraction (∼20%) of the nodes have the higher RAM needed for efficient indexed lookup (16Gb-24Gb), while the majority of nodes need only have 4Gb of RAM for the local alignments phase of the computations.

From the balanced timing profiles (see Supplemental Materials S1: Figure S12), it is not easy to make the overall process substantially faster because the indexed lookup, local alignment, and result curation/file handling all take comparable time. Thus, even if the indexed lookup were made infinitely fast, the overall running time would be reduced modestly. Therefore, dramatic overall speed improvements seem unlikely without specialized hardware accelerators or tiered algorithmic approaches. However, improvements in indexed lookup speed can be translated directly into accuracy improvement, by searching relative to a large number of indexes *without increasing* the overall running time. This will indeed become viable as bigger dynamic memory capacity becomes possible. There is, however, a limit to the strategy of bigger indexes, in that by being resistant to all possible variants by use of a much bigger index, will require longer key sizes in order to limit spurious CALs. For shorter reads, this will begin to reduce the accuracy of any one index, at which point this strategy loses its effectiveness. The optimal parameters will need to be adjusted over time as sequencing technologies produce longer and cleaner reads.

While BFAST was designed to support the resequencing of human genomes with short reads, it is in fact a completely general sequence alignment tool that should perform well in aligning any number of reads, of any length, to any target genome. Because it supports a flexible and fast indexed lookup methodology, and permits this as a user configurable feature, it should be possible to configure BFAST to work well for many large-scale resequencing problems. Even in the limit of long reads, say 100–1000 bases, where high accuracy is easily achieved with a single index, the speed-optimized indexing employed by BFAST will still provide near-optimal lookup performance.

## Methods

### Simulated Variant Classes

#### Simulation strategy

To better reflect the real alignment problems of interest, simulated reads are derived from the human genome (NCBI Build 36.1), rather than constructing an artificial random genome to permit the assessment of the sensitivity and accuracy of alignment of short reads that contain variants including errors, single base mismatches, insertions, and deletions as well as combinations. As single base mismatches are an error mode common to all technologies, we investigate high mismatch rates, as well as mismatches in combination with insertions or deletions, as might occur in reads that contain both a real variant and errors, which occurs frequently in practice. We evaluate the different variant states separately in order to obtain accuracy measures for each type of event, as they differ in the degree of alignment difficulty. We examine both the true positive rate, or the sensitivity, to assess what fraction of reads can be located back to their appropriate location, and we assess the mismapping rate, which is determined as the fraction of all reads that are mapped to the genome that are mapped to an incorrect location. Ideally, a method will maximize true positives and minimize mismapping. For these comparisons, we do not require that the exact edits (mismatches, insertions, and deletions) introduced in a simulated read be observed, but rather that the read be placed approximately in the correct location since some methods can align a read with an indel to the approximate location, but never call or specify the indel since they perform ungapped alignments. We tally the results in this manner so as to perform a more reasonable comparison between the core alignment aspects in the context of the whole human genome of other methods.

We simulated reads from the human genome by creating sets of reads with a fixed number of variants. To produce a synthetic dataset, we randomly choose an *L* letter long substring from the human genome. Each selected string was randomly altered to contain a specified number of mismatches, insertions or deletions, to produce a final read for the variant class of reads.

We generated 10,000 reads for each variant class. This number of reads was sufficient to obtain robust performance statistics.

In total, 187 different variant-specific nucleotide datasets were created:

1. Reads with exactly *x* mismatches (0≤*x*≤10).
2. Reads with one contiguous *x* letter insertion (1≤*x*≤10) and *y* mismatches (0≤*y*≤5).
3. Reads with one contiguous *x* letter deletion (1≤*x*≤10) and *y* mismatches (0≤*y*≤5).

Similarly, we also generated 10,000 50 bp variant classes in ABI SOLiD color space:

1. Reads with one SNP and *x* color errors (0≤*x*≤5).
2. Reads with one SNP, a 10 bp deletion, and *x* color errors (0≤*x*≤5).
3. Reads with one SNP, a 10 bp insertion, and *x* color errors (0≤*x*≤5).

The mismatches, and insertion or deletion break points, and color errors (ABI SOLiD data only) were uniformly distributed within the reads. For the nucleotide data, the high number of mismatches, and for the color space data, the high number of color errors, are meant to represent reads confounded by the impact of sequencing errors on both SNP and indel detection. This is especially important for ABI SOLiD color space data due to its higher error rate, which is corrected only after successful genomic alignment. Additionally, the high number of mismatches (for nucleotide data) considered might correspond to a read from a variant dense region, where several single base variants are further confounded by several read errors. Since BWA and MAQ rely on the color qualities for ABI SOLiD data to detect errors, we give color errors a color quality of 20, and 30 otherwise. The simulated datasets are available at http://bfast.sourceforge.net.

We call a read correctly aligned if the read was aligned uniquely within 10 bases of the original location. If two alignments were found with the same best score, the read was not called correctly aligned. In our simulations we do not require that the errors, SNPs, or indels be placed correctly or even found by other methods, but instead require that the read is placed within 10 bases of the true location, thus assessing global alignment rather than local alignment.

We evaluated each algorithm using one compute node, with two dual-core AMD64 processors at 2.0GHz and with 32GB of RAM. Each algorithm was run as a single process, and thus does not take advantage of any multi-threading or parallel processing capabilities of the algorithms, including those of BFAST. This comparison is done only to evaluate the relative speeds of the various algorithms under comparable hardware architecture, since many processors typically would be used in practice, which is the practical solution implemented with BFAST in practice. The precise settings for each algorithm, including BFAST, are described in the Supplemental Materials S1: *Section Algorithm Settings for Simulations*.

### Illumina Datasets

For demonstration purposes, we used a 10.9 million 36 base read data set from the human genome. In total, 33 different regions with known mutations across 5 genes were PCR amplified individually and pooled. Amplicon sizes ranged from 191 bp to 762 bp. After purifying each amplicon with QIAGEN PCR Purification Kit, the amplicons were pooled in one tube to create an equal mixture of all products. The sequencing library from the genomic fragments was prepared using manufacturer provided genomic library preparation protocol version 2.3 (Illumina, La Jolla, CA). Specifically, this dataset consisted of sequence from PCR products known to contain 13 mismatches, as well as 6 small insertions and 6 small deletions, and sequenced at a depth of coverage generally exceeding 1000-fold. We also analyze 3.5 million 55 base paired-end reads of human genomic sequence from our Illumina GAII sequencer. Libraries were generated from genomic DNA. We then selected only the first end of the pair, giving us 3.5 million 55 base pair single-end reads for alignment.

### ABI SOLiD Datasets

One million reads from two different runs of in house generated ABI SOLiD sequencer data was used for all comparisons, as this is a sufficient dataset to offer reasonable comparison. Both datasets consisted of sequences from human genomic DNA, generated by using standard 25 base and 50 base manufacturer supplied protocols.

### Support for Paired-End Data

BFAST supports paired-end data by finding CALs for each end separately. Before local alignment, the user has the option to mirror CALs for one end using the other by specifying an estimated paired-end insert length. The paired-end insert length can be inferred by examining paired-end reads for which each end has only one CAL. The option to mirror (or rescue) one end of the read can help either to improve accuracy or to use one end of the pair as anchor for the other. Each CAL for each end is then locally aligned independently. The criteria to choose the best pair of alignments for the ends are then dictated by the user, and can be based on best-combined score, uniqueness, as well as other post-alignment filtering criteria. Further details on paired-end support can be found in the Supplemental Materials S1.

### Support for ABI SOLiD Color Space

To support ABI SOLiD color space reads[21], [22], we first convert the reference sequence to color space such that each genomic read offset is artificially started with an A base to mimic the process of decoding within the SOLiD system which always generates an A terminal base in the ligated oligo in library creation. CALs are identified in color space, under the assumption that errors are more common than variants, and therefore more color errors will occur than variants encoded in color space. After finding CALs for each read, we use a modified local alignment algorithm previously described for color space reads by Homer et al.[17] and Rumble et al. [23]. This local alignment algorithm searches the space of all possible color errors, nucleotide mismatches, insertions and deletions. In this process, BFAST is able to use the entire color string for alignment.

## Supporting Information

Supplemental Materials S1 Supplemental Materials S1
(1.45 MB DOC)
