# Supplementary material for: BFAST: An Alignment Tool for Large Scale Genome Resequencing
Source: PLoS One. 2009 Nov 11;4(11):e7767. doi: 10.1371/journal.pone.0007767 (PMC2770639; doi:10.1371/journal.pone.0007767)
Supplement: Supplemental Materials S1 — (1.45 MB DOC) [file pone.0007767.s001.doc]

# Supplemental Materials S1

# BFAST: An Alignment Tool for Large Scale Genome Resequencing

Nils Homer^1,2^, Barry Merriman^2, †^, Stanley F. Nelson^2^

†Corresponding Author:. Email:[barrym@ucla.edu](mailto:barrym@ucla.edu)

1 Department of Computer Science, University of California Los Angeles, Los Angeles, California 90095, USA

2 Department of Human Genetics, David Geffen School of Medicine, University of California Los Angeles, Los Angeles, California 90095, USA

**^Contact^:** ^Barry Merriman, Ph.D. Email^: [barrym@ucla.edu](mailto:barrym@ucla.edu)^. Tel: 310825-7920. Fax: 310-794-5446^.

# Design Principles

When introducing a new algorithm, it is helpful to consider the underlying design principles and constraints. These in general include the problem that is being addressed, the limitations of current solutions, and principles underlying the new algorithm. Here we briefly outline the design principles that motivated and guided the creation of BFAST.

## Problem Specification

The practical problem is to align ~1 billion short sequence reads (25-100 bases) to a large reference genome (~3 billion bases) in a real time of ~1 day, with good accuracy for reads including sequencing errors and genomic sequence variants. The computational environment is assumed to be loosely constrained to a computer cluster of reasonable size, such as 16-64 high-end multi-processor nodes.

This problem arises from the combined requirements and constraints of whole human genome resequencing using the current massively parallel DNA sequencer technologies. The primary motivation of accurately resequencing individual human genomes with minimal false negatives for biomedical research. The diploid genome of an individual human consists of ~6.4 billion bases, and all whole genome resequencing strategies rely on a "shotgun" approach of randomly sampling much shorter reads from this pool, and to ensure that most bases are read several times for accuracy requires at least ~10 fold oversampling, or the generation of ~60 billion bases of sequence is a minimal requirement. To be useful, such short reads also must be aligned to a reference haploid human genome, ~3 billion bases in size. For technologies that provide short reads in the 25-100 bases range, this sets the "space" scale of the problem at 1 billion such short reads to be aligned to a ~3 billion base reference genome in the course of resequencing an individual human. The time scale is related to the throughput of current sequencers, which are capable of raw output of ~100 million to ~1 billion such reads at the end of a 7-16 day running period. Thus, simply keeping up with the output of one such machine would require aligning as many reads in several days, and assuming dedicated resequencing efforts will use several machines to increase throughput, human genome resequencing efforts will approach the demands of performing the full ~1 billion read alignment task in ~1 day of real time. Of course, the scope of this task scales without limit in proportion to the number of individuals being simultaneously resequenced, but we assume the functional unit will be a small number of sequencers, with a dedicated computer cluster handling alignment and ancillary sequence analysis, and this unit will then be replicated to whatever scale is necessary to handle the total number of individuals to be resequenced.

## A Brief Survey of Previous Methods

Many algorithms have been developed to perform sequence comparison, and we present a small subset of algorithms specific to our current problem, as well as some algorithms that are currently in use today. Dynamic Programming algorithms [1] are able to rapidly and optimally align a read to a target sequence with a time complexity proportional to the target size and read length in time, and read length in space [2]. This is an excellent finishing technique when the target has been reduced to a region comparable to the read length in size, but if the target were the whole human genome, this is far too slow. Moreover, even a brute force parallel computing approach is far from practical, although it is valuable to consider this just to illustrate the true scale of the problem. This task is fully parallelizable, since the total set of *N* reads and the total human genome sequence *G* can easily be subdivided over any number of processors, with the finest scale being one read aligned to a ~300 base target segment from *G*. There are ~10^7^ such target segments in *G*, and for *N* ~10^9^ reads there are thus 10^16^ subproblems. Currently these can be processed at a rate of ~100,000/second on a single CPU, which results in a total computational time of 3,170 CPU-years. Thus, a 1-million processor computer would be required to achieve the 1-day timescale. While this is at the extreme limit of current massively parallel computing configurations, this is clearly not a practical solution for the many such resequencing efforts planned.

In order to handle alignment to large targets more efficiently, many variations on the standard Dynamic Programming algorithm have been proposed to reduce time and space complexity [3], including the use of suffix trees, partial suffix trees, and Four Russians, among other data structures. For example, a suffix tree of the reference genome can be created and stored linearly in the size of the genome [4]. Nevertheless, the costs of searching such a construct for the presence of a given read, and allowing up to *M* mismatches, is exponential in *M* using these algorithms [5], and thus rapidly becomes impractical for reads that may contain several mismatches. Additionally, these approaches do not allow for insertions or deletions in the reads, which are biologically relevant variants. Therefore, standard approximate string matching algorithms, although highly developed, do not provide a practical solution.

As a direct result of the unavoidable cost of aligning to a large target, algorithms have been developed that attempt to rapidly reduce the size of the search target for aligning a given read, typically by passing it through an index of the reference genome [6-10]. Other algorithms reduce the search space by taking the opposite approach, by indexing the reads and searching the reference genome [11,12]. Using an indexing approach, all the algorithms reduce the time complexity by trading off accuracy and completeness of the search, searching for candidate alignment locations (CALs) for which local alignment is performed. Nevertheless, the large number of reads still overwhelms standard tools such as BLAST [6] and BLAT [8] in the context of human whole-genome resequencing, and the trade-offs made for speed often show up as a noticeable loss of accuracy for aligning short reads containing differences (mutations, indels, or errors) relative to the target sequence. The SSAHA application [10] can efficiently align long sequences but fails on shorter read length technologies. ELAND, an alignment tool provided with the Illumina 1G and GAII sequencers, is able to align reads with up to two mismatches, but fails to model insertions and deletions in its alignment and currently cannot be applied to longer read lengths [7]. Similarly, MAQ utilizes a the Phred-like quality score from the Illumina sequencer on base calls to better align each read [12]. MAQ indexes the input reads into memory with read lengths up to 63 bases, limiting its scalability. Similar to MAQ, SOAP models small contiguous insertions and deletions as well as mismatches (1-2 bases) using a hash look-up table of the reference to speed up alignment [9]. SOAP has a read length limitation of 60 bases and is able to iteratively trim the 3' end of the read to avoid errors at the end of the read. SOAP however does not perform a Smith Waterman alignment, thus ignores possibly relevant biological events beyond its hard limit on mismatches. SHRiMP indexes the reads first and then scans the reference genome to find the correct locations for the reads [11]. In practice, SHRiMP uses one index, limiting its ability to be tuned based on accuracy, cannot handle more than a few million reads at time, limiting the compression benefits of indexing the reads, and suffers from poor practical running times. In aggregate all of these current algorithms still suffer from a variety of shortcomings, including limited performance or accuracy, ignoring biologically relevant events (>3bp insertions, deletions or >3 mismatches), or limitations on read length or the ability to process a large number of reads at once. Furthermore, they do not explicitly include an *a priori* method to assess the accuracy of alignment.

It is interesting to note that in general, algorithms that index the reads rather the reference will have sub-optimal performance. Furthermore, it is only the case when the number of bases total present in the reads exceeds the number of bases in the target, which is the case in the whole genome resequencing, does the idea of indexing the reads become theoretically more attractive, since this technique would in effect compress the read data to only search over unique reads. However, in this strategy for every experiment (set of reads), a new index and indexing strategy must be created for the specific set of reads. On the other hand, for experiments that use the same reference genome, one index of that target can be created and re-used for each subsequent alignment run, incurring no overhead beyond the first index creation for each additional experiment. This is likely the preferred general strategy as many human genomes will be generated by the current activity of the community. Other practical limitations on read indexing include the need to first sort the reads, or at least have a means of tracking all possible *k*-mers originating from that read. Thus, if a lookup in an index of a reference genome is approximately constant in time, then whenever a read was analyzed (i.e. performed even one comparison), we could have instead looked up this read in the index of the reference genome and found its candidate alignment locations.

## Limitations of Earlier Methods

Our purpose here is not to comprehensively survey all sequence search and alignment algorithms, but rather simply to illustrate how algorithmic choices that were appropriately made for previous large scale alignment challenges can be sub-optimal for the new problems of error prone short sequences, and indicate where improvements are possible when designing a new algorithm. The BLAT program, [8], is an illustrative example of a successful and widely used search and alignment tool that was optimized to meet the challenges presented by the Human Genome Project and its aftermath, but has not been optimized for the present problem of massively parallel genome resequencing. Nonetheless, BLAT is useful for this purpose, and our own development presented here was in part motivated by a year of experience using BLAT as the alignment engine for aligning several billion reads from a pair of Illumina/Solexa sequencers operating in our lab in an effort to permit identification of biologically meaningful short insertions and deletions.

Let us briefly consider the specific design choices made in BLAT, and how some of these reduce its effectiveness for the current problem. BLAT uses a two stage approach to align a given search string (here the "reads") to the reference genome: first it produces a list of candidate alignment locations for the string, based on a fast heuristic "search", and then it employs a standard rigorous local alignment algorithm for each of these candidate locations, recording the optimal alignment and related score. The speed and accuracy, the ability to include the correct alignment among the candidates, of this approach are primarily dictated by the search phase. In BLAT, the fast search is done by looking up all short *k-*mer words found by scanning across the search string (for a string of length *L*, *L-k+*1 choices, e.g. 20 for a *L*=30-mer read and *k*=11-mer words) in an index of *k-*mers in the genome. If a *k*-mer from the search string contains a variant relative to its true position in the reference, it is not directly useful for indexed look-up, and in this way variants impede the look-up process, and will render it totally unsuccessful if they occur in sufficient number and distribution in the search string. In order to make the lookup robust against such variants, BLAT favors smaller *k*-mers (typically *k*=11 for DNA) but requires multiple hits in the index (typically two) that are independent but nearby in the genome in order to reduce the number of spurious candidate locations. For example any given 11-mer would occur in thousands of places genome wide, but two nearby, non-overlapping 11-mer hits approximate a 22-mer lookup constraint, which would typically be unique in the (non-repetitive) human genome (see Figure S2 and Figure S3). Finally, it is critical that the entire index reside in the dynamic computer memory for fast look-up, and in order to reduce the size of the index of a human-scale genome to fit in the dynamic memory constraints of the time (~1 GB), instead of indexing all k-mers in the genome, only the subset of *non-overlapping* *k*-mers were indexed, thus making the index memory requirement *k* times smaller than it would be otherwise. Yet for the present problems, several of the above BLAT design choices result in practical inefficiencies. The number of reads to be aligned in our scenario is much greater, and even with improvements in computer speed in the intervening years, algorithmic speed increases are essential.

In regards to speed, BLAT looks up short *k*-mers, each of which may produce a large number, *N* ~100-1000, of genome locations per index hit, and then these hit lists must be merged in order to determine if two *k-*mers occur near enough to identify a candidate location. This merge is at best an O(*N*) operation process (actually O(*NlogN*)), so there is a factor of ~*N* in speed that is lost by not having unique locations associated to each entry in the index, as would be achieved by indexing longer words.

Next, in regards, to accuracy, BLAT strives to minimize the size of the index to fit memory constraints by not indexing all *k*-mer words in the reference, but this results in more index lookup failures and thus a potential loss of accurate placement. This becomes important for looking up short strings that have only a few *k*-mers that would appear in the reduced index, so that the lookup can fail due to just a few variants in such a short string.

Finally, there is the overall indexing strategy: using a *single* *k*-mer index from which we require *m* nearby hits, leads to a (*k*, *m*)-parameterized search strategy that provides comprehensive but not very flexible control over the speed and accuracy trade-offs. The only ways to be more robust against variants are to decrease *k* or *m*, but downward increments in these cause rather rapid (exponential) growth in the search time or in the yield of spurious candidate locations. For BLAT's original problem domain there is a range of (*k*, *m*) that yields acceptable speed with high accuracy, but for the present problem of many more, and much shorter reads, these parameters no longer provide for fine control over the speed and accuracy trade-offs inherent in the approach.

## BFAST Design Principles

We now consider the general principles that guide the design of the present algorithm for fast and accurate short read alignment to a reference genome. The general procedure, as for BLAT, is to have a fast indexed lookup phase to produce candidate read alignment locations, and then a second local alignment phase using a standard optimal local alignment algorithm, so that the speed and accuracy burden mainly rests on the index phase. In that regard, we are guided by the following principles intended to favor speed and accuracy:

1. Index the genome as heavily and completely as possible, using longer search keys that favor unique locations, so that mapping reads approaches the ideal of looking up a unique location. This amounts to maximizing the general speed-space trade-off for this problem in favor of speed at the cost of space.
2. The basic look-up process for a key in the index should be essentially an O(1) operation, so that the speed advantage of an extensive index is not offset by the cost of the lookup procedure.
3. Greater accuracy is to be achieved by using multiple indexes based on different masks to define the index keys, but keeping the number of letters in the key, *k*, large for uniqueness. Avoid using shorter keys (reducing *k*) to obtain accuracy, which results in exponential growth in spurious candidate locations.
4. Keys should be kept as short and compact as possible while still providing a high degree of uniqueness in the positions returned from a lookup, since longer keys that include or span more bases of sequence conflicts with accuracy in the presence of read variants.
5. Each whole-genome index is costly to read into dynamic memory, so each index should be read in just once, and then used exhaustively, before resorting to additional indexes.

Let us first highlight the departures that this suggests from the BLAT algorithm: BLAT intentionally reduces the index size, sacrificing completeness for reduced storage, which contradicts the first principle. The BLAT reliance on short words that index to multiple locations, combined with a costly merge process to obtain unique locations goes against both first and second principles. BLAT achieves accuracy by reducing the *k-*mer word size, in contrast to the third design principle. The latter two principles, in contrast, are somewhat implicit in the formulation of BLAT. Instead, adhering to these principles leads rather directly to the algorithm used in BFAST, which we briefly outline here, and then discuss in more technical detail in the subsequence section.

### Index Class

First, we introduce a class of indexes that allow us to index the genome more extensively, as suggested in the first and third design principles. Instead of indexing the location of *k*-mer words in the genome, we generalize this concept to indexing the start positions of *k*-letter substrings that are obtained from a mask, which is slid along the reference genome at one base shifts to generate the index data. This is similar to spaced seeds introduced previously in homology search programs [13-15]. For example, the letter selection mask suggested by the bit-pattern 0011001010, directly applied to the sequence "AAGATTACAG", selects the letter key "GAAA". For the sake of unambiguous terminology, we refer to the *key size*, *k*, of the mask as the string length of the index keys it defines, i.e. the number of bases it selects, and the *width* of the mask as the number of positions from its first selection point through its last selection point. Thus, the above mask would have a key size of 4, and a width of 7---note the width ignores any padding 0's on either end since for our purposes masks identical by translation are equivalent, and thus padding 0's are irrelevant. In the spaced seed terminology, we have key size being the weight and the width being the model [13-15].

A contiguous *k*-mer is one such mask option (both the key size and width equal to *k*), but typically a mask also spans bases that are not utilized in the key, which is what provides robustness against sequence variants. For a mask width *w* > *k*, there are *w*-2 choose *k*-2 different masks, each leading to a different index of the genome (except for masks identical by translation). In particular, for reads of length *L,* there are *L* choose *k* potential masks upon which to build indexes for looking up such reads, which is a huge number of options in the cases where 25 ≤ *L* ≤ 100, and 18 ≤ *k* ≤ *L*, for example. We will generally use a small number of such indexes, chosen to be sufficiently robust against the anticipated number of variants and sequence errors that may occur in the reads, but it is fundamentally this diversity of masks with the same key size *k* that allows us fine control over accuracy versus lookup speed.

#### Fast Index Lookup

Second, we must consider how to efficiently look up a given *k-* letter key in such an index, as per the second design principle. Note that such an index of *k*-letter keys scanned from the genome will include only a very sparse subset of all 4*^k^* possible keys, whenever 4*^k^* is much greater than the size of the genome, or *k* > 16 in the Human case.

It would be extremely space inefficient to store this index as a list corresponding to all 4*^k^* possible keys, as most list entries would be empty, not corresponding to any position in the genome. Thus, the index cannot be stored explicitly in a way that simply permits immediate translation from a given *k-*mer to its position(s) in the lexically ordered table of all *k-*mers. In contrast, this simple representation would provide an optimal space and speed efficient lookup strategy for smaller *k*  (*k* < 16), such as that used in BLAT. A naive approach for large *k* > 16 would instead store the actual *k*-mer keys that are observed in the genome in lexical order, and search through these keys using bisection to look up a given key in the index, much as one might do in looking up a word in a large and unfamiliar dictionary. This approach violates the second principle, however, as we would consume a substantial fraction of time in the look-up procedure itself. What is needed is a suitable "index to the index", or hash table, which allows us to rapidly lookup up a given key based on pre-tabulated information.

Taking a cue from large dictionaries, which provide thumb-tabs to rapidly identify where to find the words that start with "A", "B" ,"C", etc., we index all *j-*mers as they occur in the lexically ordered list of keys, for some *j* that is small enough to tabulate exhaustively for all 4*^j^* possibilities. For each *j*-mer, we list the first and last entry location in the index of keys that start with that *j*-mer. When looking up a given *k*-letter key in the index, we look up the first *j*-mer of this key in the hash of all 4*^j^* ordered *j*-mers, which is immediate, and then given the possible start and stop locations of our key in the index, we perform as much bisection as necessary to locate the key of interest. In practice, for *j* that are large but manageable(e.g., *j*=14-16), there is little bisection needed in most cases, and we can look up our keys that are a sparse collection of long *k*-letter words (e.g. *k*=18-22 in practice), with essentially the same O(1) cost required to compute the position of a *j*-mer in the lexically ordered list of all *j*-mers. More precisely, this hash lookup amounts to evaluating the *j*-mer, viewed as a base 4 number, with the quaternary digits being the DNA base codes "A", "C", "G", and "T".

#### Efficient Index Storage

Finally, to complete the specification of an efficient index look-up procedure, we note that the index keys must be available in some fashion, but it would be space inefficient to explicitly store all the *k*-letter keys found in the genome, as this would amount to storing the letter content of the genome *k* times over. Instead, we use the far more efficient representation of storing just a single copy of the genome, with each key represented by the start location of the mask in genome, combined with knowing the mask itself. This in effect stores the keys of the index in an efficiently accessible and nearly optimally compressed format, at the cost of storing one copy of the reference genome sequence. This amounts to a minor overhead for key storage, since the genome storage requirements are ~10 times smaller than the size of an index in the case of the human genome. A similar relation is true in general, since for a genome of *G* bases, storing the sequence of the genome requires *GB_l_* bits, where *B_l_* is the number of bits used to encode a single letter (e.g. take *B_l_* =2 for the DNA base codes and *B_l_*=4 in order to include the letter "N" specifying an unknown base), whereas the content of the index is essentially all positions in the genome---suitably related to keys---which thus requires *GB_n_* bits, where *B_n_* is the number of bits needed in the binary integers required to enumerate the genome, (e.g. *n*=32 for Human), *n*=log_2_(*G*). Thus, the minimal index storage requirements exceed the minimal genome storage requirements by the factor log_2_(*G*)/2, which is generally on the order of 10, making this a generally efficient key storage strategy.

#### Algorithm Outline

The general algorithm used by BFAST is set as 1) index the locations in the genome of specific *k*-letter keys according to some set of masks, with *k* large enough to give essentially unique locations (typically *k*=18-22), and 2) for each such index, use a hash table of all *j*-mers to efficiently look up keys in this index, typically *j*=14-16, as described above. In applying a given index for each read, keys are generated by shifting the mask across the read at a given shift spacing (typically taken to be one) and all such shift keys so obtained are looked up to potentially provide candidate alignment locations. This is carried out for each index, with the resulting cumulative collection of candidate alignment locations passed on to the second phase, which consists of a local Smith-Waterman optimal alignment.

All that remains is to specify the actual indexes for a given problem. Typically, more than one index is needed to achieve good accuracy in the face of variants and errors within the human genome, although as read lengths become longer, exceeding 100 bases, a single *k*-mer index may suffice. Note that even in this longer-read limit, while we would have a BLAT-like index, relative to BLAT we would still have a more accurate index, since it would include *all* *k*-mers, and we would still be using a much more efficient look up strategy, due to the much larger key size, *k*.

### The Impact of Memory Limitations

As an important practical matter in our implementation, it is possible to store an index for the human genome, its associated hash table, and the whole Human Genome sequence in ~17 GBs of dynamic memory, so that BFAST generally runs optimally on a node with several GBs more than this, to allow for added system and program overhead. At present, 24 GBs are ample, but if there is inadequate memory to index the whole genome, the BFAST program can split the genome into indexable chunks based on the user-specified available memory, and process these serially, to fit within the available dynamic memory. However, note that this is less efficient, as each read is re-processed for each portion of the index, so the time spent on indexed lookup increases accordingly. The fundamental design principle is to index as heavily as possible, and this is ultimately limited by the practical constraint that an index must fit entirely into dynamic memory for fast access. Just as the design of BLAT was impacted by a ~1 GB dynamic memory limit at the time, the design of BFAST is also impacted by the current dynamic memory limits, which are ~32-64 GB for the current class of high-end cluster nodes. It is worth briefly considering what memory levels would be needed for improved index performance, as they will undoubtedly become feasible in the next several years. As noted, at present each human genome index requires ~17 GB of dynamic memory (1.5 GB for the genome, 14.5 GB for the primary index, and 1 GB for the hash index). There are several memory levels that could improve index performance in major ways. First, each read must be looked up in forward and reverse complement orientation in the reference genome, and we presently do this as two separate look-ups for the read. However, if 33 GB of memory were available for storing indexes, we could simply index the forward and reverse complement of the reference genome as one large genome of twice the size, thus cutting the overall lookup time in half. For the special case of bisulfite sequencing to read the methyl "C" base, the process converts all "C"s to "T"s, so the forward and reverse strands convert differently, and thus in turn have different reverse complements, so that in total there are 4 genomes to be indexed, and this could be performed in one comprehensive index that is 68 GB in size. More generally, and more demanding, a very powerful index would be to include one-mismatch matches using a key size of *k*, so that a single base error or variant would be automatically factored in when looking up each *k*-mer key. This index is 3*k* times larger than a standard index, since each *k*-letter word in the genome spawns 3*k* one-mismatch possibilities that index to the same location. Thus, such an index for *k*=20 would require ~1 Terabyte of dynamic memory. Such an index would be very robust against errors and variants, and it is likely a single index would suffice for many purposes, making the lookup process several times faster than at present, and/or more accurate. While this may seem extreme, nodes with 256 GB are already commercially available, so it is likely to be possible within a few years.

In this same spirit, but much more practical, for a given sequencing technology not all errors are equally likely, and indeed often for a given base, it is only likely to be confused with one other base, rather than three. Thus, we could imagine indexing only the likely error modes for the given *k*-letter keys, so that a given key will automatically index to not only its occurrences, but also to the occurrences that are likely errors when reading that key. This may expand the index only by *k* rather than 3*k* for all off-by-one errors, so that we can index a *k*=20 letter read in this fashion with ~330 GB of memory, although this becomes technology dependent, and its accuracy can only be estimated in a probabilistic sense, based on the probability of the various read errors occurring. These space estimates ignore the hash size, which may also be required to grow in size if the index is larger. For example, quadrupling the index size may also require a quadrupling of the hash size so that the expected look up time of a key in the index is still O(1). These examples illustrate that there are still indexing performance gains to be had as larger dynamic memories become widely available, which could be used to provide practical--but not dramatic--speed or accuracy gains for future versions of BFAST.

# Supplemental Methods

In this section we describe our algorithm and implementation in more precise and formal detail. The alignment algorithm consists of a two-part procedure: in the first phase, for each of the *N* reads we find a set of Candidate Alignment Locations (CALs) in the reference genome. The CALs are obtained by lookup using a series of whole-genome indexes chosen to provide robustness against errors or variants in the reads. In this phase, for each index, every read is processed, and the CALs are accumulated index by index until all indexes have been queried and the total set of CALs accumulated. In the second phase, a standard Dynamic Programming method [1,16] is used to produce optimal local alignments for each CAL.

In the case of ABI SOLiD data, we employ an extension of the local alignment algorithm that can simultaneously decode and align the read in color space to a nucleotide reference sequence [17].

## Index Construction

We first begin by describing the structure of an index we use to search for CALs. Each index is based on a selection mask, which defines the substring keys for the index. We can represent a mask as a series of zeros and ones, where the bases to include in the index key are identified by a one, and those to be omitted by a zero, when the mask is place over a given sequence location. Given a mask, the index is created by shifting the mask across the reference genome, at one base increments, and associating the mask position with the key defined by the mask at that position. When this shift process is complete, each distinct key observed in the genome will be associated with a list of all genomic positions where that key occurs, and the lookup process for any given search key corresponds to obtaining the associated list of positions from the index. For terminology, we call the number of positions from the first 1 to the last 1 the *width*, *w*, of the mask, while the total number of 1's is the *key size*, *k*, of the mask, and the *k*-letter substrings defined by the mask are the keys. As a convention, we restrict masks to begin and end with 1, for definiteness in determining which shifts of the mask are valid. This mask together with the hash width defines the layout of the index. Regarding the lookup process, for a given read of length *L*, by shifting the mask in one base increments across the read, we obtain a total of *L*-*w*+*1* possible keys that can be used to lookup up CALs for the read from the index. Due to the size of an index and the desire to be robust against variants or errors within the read, using most or all of these available keys is generally beneficial. Within BFAST, it can be specified which of these shifts or offsets to use for lookup keys for the reads, although according to our design principles we use the index most efficiently when all possible offsets are used.

For a few examples, a mask that uses 25 contiguous bases to form each key would be *m*=111…111, which has a width *w*=25, a key size *k*=25, and the layout description consists of the single tile of length 25. Or, at the other extreme, a mask that utilizes every other base of a 50-base region to form a key is *m*=101010…010101 which has

a width of *w*=49, a key size of *k*=25. In general, in this manner, any arbitrary subset of the read can be used or omitted when searching for CALs by using the appropriate mask.

The index set employed will control the accuracy of finding the correct CAL for reads that contain errors or variants. For a given read, if a key formed by the mask contains a variant base, the index lookup will fail, and if the mask straddles a breakpoint in the read (corresponding to a deletion or insertion) the lookup will also fail. Thus, the criteria for successful read lookup---i.e. obtaining a CAL that is the true position of the read---is that at least one of the index masks will have a shift position on the read that does not straddle any breakpoints, and does not contain any base variants in the selected key. This favors a set of indexes from masks that are suitably independent in the bases they select, as well as those with a smaller width, to avoid straddling breakpoints. For completeness, the BFAST distribution contains tools for assessing and selecting good index sets, including a collection of optimized index mask sets for read lengths from *L*=20 to *L*=100, which were chosen by a random search optimization method, as described in more detail in a later section.

### Index Data Structure

Finally, we introduce a novel data structure that is the basis for the BFAST implementation of these index features. This structure guides efficient implementation, and is useful conceptual device in its own right. This structure is illustrated in Figure 1 and is based on the following motivation. Since indexes keys are meant to be long *k*-letter keys, it is conceptually helpful to think of them as all being derived from a single universal master table that consists of all the longest possible keys in the genome, i.e. the maximally long strings from the genome reference sequence. That is, at each position in the reference, we consider the *suffix string* that extends from that location to the end of the genome, which is the most comprehensive key that could ever be associated with that location. (In practice, the suffix can only extend to the end of the present contig, meaning the end of the chromosome, or to the first gap or "N" in the sequence, and for suffixes starting near such ends, some "boundary condition" is required, such as ignoring suffixes shorter than the read length of interest.) The list of all such suffixes---or maximal keys---is a conceptually useful master table of strings in the genome, although actually storing it explicitly would be impossible. However, it can be stored in a compact, trivial way simply as the list of genome start locations 1, 2, 3, …, *G* of the suffix strings, in conjunction with the stored genome sequence itself to obtain the actual strings. The specific indexes we construct can then be thought of as placing the mask at the start of each suffix string, and lexically sorting them according to just the mask-selected bases, a reordering that is again always stored in the compact form of the reordered list of suffix start locations. The compactly stored forms of the master suffix string table are the basic index data structure implemented in BFAST, along with the associated hash of location ranges in these compact tables, and is illustrated in Figure 1 of the main text.

## Searching for Candidate Alignment Locations

In general, the search for Candidate Alignment Locations (CALs) consists of looking up keys---derived from the reads--in the various indexes of the reference genome. However, within this general CAL search process, the BFAST algorithm makes use of several strategic points to achieve additional performance improvements in speed or accuracy. These finer points of the CAL search process are outlined in this section.

To accelerate the CAL search, we utilize a special pre-filtering lookup step. The concept is that many of the reads will be free of errors and variants, and thus map perfectly to the reference genome (although perhaps to multiple places). These should be rapidly identified and excluded from the multiple index searches used for robustness---even if such searches did produce additional CALs, the resulting non-perfect alignments would never be favored over the available perfect alignments. This short cut reduces the overall search time at very little added cost, especially for the shortest reads (~25-mer) where over half the reads may map exactly to the reference.

To implement this pre-filter in a more general fashion, we adopt a two-tiered CAL lookup strategy, using a special primary set of indexes and a secondary index set. In the first tier, we apply the primary index set, and if at least one CAL is found for a given read, that read is set aside for direct passage to the optimal local alignment phase of BFAST, and is not tested against the secondary index set. Only the reads that have no CALs in the primary set proceed to use a set the secondary index set to lookup CALs.

In practice, the primary index set is meant to approximate exact lookup of the entire read, and so is typically a single index derived from the mask *m*=111,…,111, which selects contiguous *k*-mer keys for a large *k*, where *k* is either the full read length, or, for longer reads, at least much longer than the length required simply for unique lookup in the genome, e.g. *k* ~35. The secondary index set instead consists of indexes chosen to be robust against insertions, deletions, mismatches and errors in the reads, and these will tend to use a smaller, but still highly unique, key size, e.g. *k*~20. The choice of secondary indexes is where the major speed and accuracy trade-offs of BFAST are determined in any given application, and this will be discussed in more detail in a later section.

### Extended Enumeration of Keys

Since indexes for the Human Genome are large (~17 GBs), loading many indexes into memory requires a significant amount of time. Therefore, when an index is loaded into memory, it is beneficial to fully utilize the loaded index before incurring the penalty of loading another index. For this reason, to improve accuracy in the CAL search, we prefer to use all possible keys generated by shifting a given mask across the read, rather than a large number of indexes with a smaller number of mask shifts per read. In BFAST, we give the option of further extending this strategy in a novel way, to extract even more useful CAL lookups from the same index. This extension helps only for reads that contain a small insertion or deletion, but these are otherwise especially difficult to lookup as the breakpoints effectively divide the useful read length. To further increase accuracy against such events, we can enumerate all small disruptive indel events that could have occurred in a read,[13-15] *which could still be looked up with the current index*, and attempt to use the corresponding keys to obtain CALs. This amounts to modifying the way the mask is applied to the read, while still using the same index. For example, if the index mask were "101" and a read were "ACGT", the same index could also be used to look up each of the possible pre-insertion forms "AGT","ACT", and each of the possible pre-deletion forms "ANCGT", "ACNGT", "ACGNT". The interpretation is that these pre-forms are what appear in the reference genome, and the read in question contains the alteration, either as a read error or a variant. Notice how the possibilities for a pre-deletion must reside within a gap in the mask, while the possibilities for a pre-insertion form is constrained only by the width of the mask. The enumeration for a hypothesized single indel of up to a fixed size will produce a quadratic increase in the number of searches to be performed---proportional to the read length and indel size, and depending on the gap structure of the particular mask---and can helpful for aligning short (<36 bases) indel-containing reads. This capability is provided as an optional "extended index search mode" of BFAST, but, as the simulations below show, for longer read lengths simply including a minimal width mask among the set of masks used provides substantial accuracy against indels.

### Repetitive Keys and Repetitive Reads

Highly repetitive sequence elements in the genome can result in the generation of many spurious CALs. Such sequence will typically be represented not only at many genome locations, but also within many reads, resulting in overall quadratic growth in the total number of spurious CALs found for a read set. This could easily dominate the total running time due to the increased number of total local alignments required. For example, in the Human Genome, there is an 18-mer from the conserved portion of the *Alu* repeat family that appears ~10^6^ times in the genome, and each occurrence of this as a key would thus yield 10^6^ CALs, compounded by the fact that it accounts for roughly 1% of the total genome sequence, and thus will appear in a similar percentage of all reads sampled randomly from the genome. Unchecked, for a billion read set this could result in ~10^12^ spurious CALs, which would render the overall alignment computation unfeasible both in time and storage requirements---all tracing back to this one repeated 18-mer.

Such highly non-unique elements carry no useful information as primary indexing keys, so to guard against this pathology, during searching for CALs it may be beneficial to exclude any key for which the number of CALs exceed a specifiable limit, *K*.

Similarly, we can use an upper limit *M* on the total number of CALs that a valid read is allowed to accumulate across the multiple indexes and offsets used. Any reads exceeding this CAL limit are annotated as repetitive, and no CALs or alignments are returned for them. The first upper limit *K* will select for more informative keys, while the second upper limit *M* will avoid performing local alignment on any read for which there are too many spurious CALs.

We performed simulations to assess the impact of *K* and *M* on the ability to include the correct CAL in the set of CALs for a read. We simulated 1,000,000 perfect match 36 base-pair reads from the Human Genome. Using a single index with a contiguous 18 base mask (*k*=18 and *w*=18), we found the set of CALS for the simulated reads using various values of *K* and *M*. In Figure S4 we plot the percentage of reads with the correct CAL included in its CAL set versus both the parameters *K* and *M.* We see that regardless of the value of *K*, a value for *M* of 32 or greater was associated with the correct CAL included in the CAL set 80% of the time or greater. We also see that the value of *K* modestly affected the rate of inclusion of the correct CAL in the CAL set. In general, it is our goal to minimize the number of CALs found while maximizing the probability or rate of inclusion of the correct CAL step. The former gives us a faster running time due to the fewer number of local alignments performed and the latter increases accuracy by reducing false-negatives. It is also interesting to observe the large increase between *M=8* and *M=16*, which is attributable to the inflection point in the complexity of the Human Genome.

## Paired End Candidate Alignment Locations

BFAST provides native support for aligning paired end reads. We outline the algorithm briefly here, which is built upon the basic BFAST lookup and alignment procedures; complete details are available in the documentation with the BFAST distribution.

There are a variety of molecular strategies for generating paired end read data, and a variety of reporting formats for the various sequencing instruments, but BFAST assumes each paired end read will ultimately be presented as a two reads converted to the same orientation and on the same strand. Using the given index set and algorithms as described, BFAST generates CALs for each end. Note that highly repetitive ends will have no CALs at this stage, due to the CAL limit F_max_, and ends with too much variation, i.e. indels and mismatches, also may have no CALs. For reads for which both ends have CALs, BFAST performs local alignment on each CAL for each end separately. This choice of the paired alignments is separated into an additional program and further information can be found in the documentation with the BFAST distribution.

Note that reads where both ends lie in highly repetitive sequence, i.e. the number of CALs exceeds the limit at both ends, will not be aligned by this process. While these could in principle carry information about breakpoints, which join large, repeat elements, in practice it will be quite difficult to reliably make use of such reads, due to the high level of ambiguity. In any case, BFAST annotates them as unaligned, so they can be set aside for subsequent specialized analysis. This illustrates a general aspect of paired end data, that additional analysis strategies beyond the primary alignment may be useful for extracting the more cryptic breakpoint information. We have found that trying to infer the alignment of one end from the other results in false-mapping, while not recovering a significant amount of mappings, especially for the human genome. This is due to the difficulty of resolving local repeats.

## Designing Index Sets

### Choice of Key size

One major component of designing indexes is choice of a suitable key-word size, *k*. A smaller *k* requires fewer bases from the read to match the reference, and thus improves accuracy, but at the cost of more spurious CALs---i.e. CALs due to random *k*-letter matches in the reference genome. Choice of a reasonable *k* balances these two trade-offs, but keeping the number of spurious CALs small is a critical consideration, as their number grows rapidly as the key size (*k*) decreases. Thus, we first briefly consider how many CALs to expect in general. We follow the analysis given in Kent [8] adapted to the present case: Let *w ≥* *k* be the width and key size of a particular mask used to build an index. The genome of size *G* will generate *G*-*w*+1~*G* keys for the index. A given read of length *L* will provide *L*-*w*+1 keys for lookup. We assume *w* is not much larger than *k*, so that *L*-*w*+1 ~ *L*-*k*+1, for simplicity. Assume all letters occur randomly and with equal frequency in the genome, so that the probability of a random *k*-letter key match is (1/*A*)^k^, where *A* is the size of the symbol alphabet (*A*=4 for DNA). Then, assuming independence of all trials, the expected number, *F*, of "false" random key matches in the index for the given read would be :

*F* = (*L* - *k* + 1)(*G*/*A^k^*) (equation S1)

For a case of practical interest, take *A*=4 for DNA, read length *L*=50, and *G*=2·3.2·10^9^, corresponding to the forward and reverse complement of the Human haploid genome reference. Then, for example, the key size *k*=11 used as the default in BLAT would here result in *F* = 61035 expected spurious locations. In contrast, for BFAST with a typical key size of *k* = 18, we have *F* =3 spurious locations per index used, and using *k* > 18 reduces the number of spurious CALs to less than one per index. More generally, in Figure S1 we see the number of spurious CALs, *F*, plotted against the key-word size, *k*, for a variety of different genome sizes. As evident in the formula and figure, the expected number of CALs decreases exponentially in key size. For larger genomes, we favor indexes with key size *k* = 16-20, so as to achieve on the order of one or fewer spurious CALs per index, while otherwise keeping *k* small for accuracy. There are two possible exceptions to this general guideline. First, for shorter read lengths, *L* ≤ 32, a smaller key size may be desirable for reasons of accuracy, which is discussed below. But such a reduction comes at the cost of 4 times more spurious CALs for each one base reduction in key size, which rapidly becomes costly in both computational time and storage. Second, the spurious CAL estimate in Equation S1 is only valid for the relatively unique part of a genome, which is well modeled by the assumed randomness. For the highly repetitive part of a genome, the number of spurious CALs depends entirely on the details of each repeat family, and it may be that using a key size *k* ≥ 22 provides a substantial advantage in removing alignment ambiguities within such families, although, again, this must be balanced against accuracy considerations. As an example of dealing with these issues more thoroughly, in a later section we study the effect of the choice of key size on spurious CALs for the Human Genome, based on the actual reference sequence.

#### Choice of Mask Layouts

Given a reasonable key size, *k*, the problem becomes that of constructing a "good" set of mask layouts for the *k* positions to be selected. Here "good" performance means successful lookup of as many reads as possible, in the presence of variants between the reads and the reference. For each mask, a given read from the genome will be looked up successfully if some translation of the mask along the read selects bases that do not differ from the corresponding locations in the reference genome. Specifically, this means that the mask cannot straddle any insertion or deletion breakpoints, and its selected positions cannot contain any single base mismatches. (We do not count as success the possibility that a combination of indels and mismatches just happens to allow successful lookup for a specific read sequence, while violating these general rules.) Each individual mask has a limited capacity to "avoid" the variants in a read in this way, so the overall set of masks should be maximally "complementary" in this regard, so that at least one mask does "catch" a valid part of a given read.

For simple design challenges, such as a mask set that is robust against any possible single base mismatch, or any single deletion breakpoint, it is relatively easy to design suitable masks "by hand". For example, if a 36-base read has a single deletion breakpoint, or a single mismatch, it is easy to see that the mask that selects 18 contiguous positions, *M* = 111…111, *k*=*w*=18, will always be able to index the read to its proper position, no matter where the breakpoint or mismatch occurs. However, to be robust against more complex constellations of mismatches and breakpoints---even as simple as a read containing both a deletion and a mismatch---a systematic approach is needed to search for good mask sets.

Thought of as an optimization problem, the abstract goal would be to achieve a specified level of accuracy for a minimal computational cost. Here, the ultimate notion of accuracy should be related to the sequence coverage generated, i.e. the fraction of possible coverage that is realized by looking up read positions from the index set. The ultimate notion of computational cost would be the time required to extract that coverage. To make the problem more concrete and tractable, we simplify accuracy to mean the fraction of reads that receive their correct location among the CALs generated from the index set. Further, we break this accuracy down according to the number and type of variants in the reads, as mismatches, deletions or insertions, since these different variant states strongly impact the success of index lookup. Also, for simplicity we measured the cost of the computation in terms of the number of indexes in the set, rather than the more desirable---but difficult to estimate---true execution time. The two are not the same, in large part because masks with smaller width generate more keys per read than those with larger widths, and as a result require more individual key look ups and may generate more spurious CALs.

Thus, the more tractable optimization problem becomes that of achieving a given success rate on looking up reads with a particular number of mismatches or indels, using as few masks as possible. For example, we may want to find a mask set that has successful lookup for at least 95% of all 50-base reads that contain 5 mismatches. In general, the result is a discrete and difficult optimization problem.

To obtain solutions to this mask design problem, we have developed simple random search strategies, and these work well for practical purposes. We will briefly outline the approach here, but the tools to perform these optimization searches are included in the BFAST distribution, as are a comprehensive set of optimized mask designs suitable for read length from *L*=20 to *L*=100 bases.

#### Assessing Mask Accuracy

First, let us be clear about how to measure the accuracy of a mask for aligning variant reads. We categorize the read variant status explicitly, as a read of length *L* containing some number of mismatches, *MM* ≥ 0, and some number of breakpoints, BP ≥ 0, resulting from a deletion (1 breakpoint) or an insertion (2 breakpoints) occurring in the read, and for an insertion, we also need to explicitly consider the insert size, *i*, since this strongly impacts the lookup capability. Given a variant specification, {*L*, *MM*, *BP*, *i*}, we can consider all such ways these variants could be configured in the read, and for each such configuration we can determine whether a given mask can successfully lookup that variant, i.e. at least one of the keys obtained by sliding the mask along the read is free from mismatches and does not straddle a break point. Thus, either by checking all such variants, or by random sampling in the case where there are too many to exhaustively check, we can compute the accuracy of the given mask as the fraction of all such {*L*, *MM*, *BP*, *i*}-variant reads with successful lookup. This makes it clear that the accuracy of a mask is actually a multi-dimensional accuracy table of these specific accuracy measures, which indeed vary dramatically across the different {*L*, *MM*, *BP*, *i*}-variant classes. This same accuracy table concept readily extends to a set of masks, as well, and this is the formal expression of accuracy we use for a mask set. For an example of this, see in the many tables presented below the mask sets and their associated accuracy tables.

#### Mask Search Strategies

We can now outline our random search strategies. In the simplest strategy, we perform a global random sampling search: we fix the total number of masks in the set, *S*, and fix the key size *k*, and we allow a search range of mask widths, extending from *w*=*k* to some upper bound. We initialize the search with some given set of masks, which we typically take to be a single mask, the most compact mask M = 111…111 of width *w*=*k*. This is a particularly useful mask (and the basis for the BLAT index, as well) because it is the most robust against indels, i.e. it is least likely to straddle a breakpoint. Insisting on this mask thus biases the mask set towards being more robust against indels, but a different starting set could be used to impose other such preferences. The remaining masks *S*-1 of the set are then sampled at random, as follows: for each we choose a width, *w*, at random from the allowed range of widths, and then we choose the mask layout, from the *w*-2 choose *k*-2 ways of distributing the *k* ones of the mask in the *w* positions, insisting that the first and last position be one. Given a full mask set, we evaluate the accuracy table. We repeat this global random sampling of mask sets for a specified numbers of samples, and retain the mask set that has the "greatest" accuracy. The result is an optimized *S*-mask set. Note the procedure requires the notion of "greater" accuracy, which means we must specify some definite ordering on the accuracy tables. The ideal ordering would be to collapses the percentages in the table into a net read accuracy percentage, properly weighting the {*L*, *MM*, *BP*, *i*}-variant accuracies by their probability of occurring in a read. Since the necessary weighting probabilities are not known, we employ a very simple approximation, assuming the most likely variant modes are mismatches, and so we judge the accuracy by using just the accuracy on mismatches, the most accurate mask set being the one that has the greatest cumulative mismatch accuracy. Because this measure ignores the accuracy on indels, we attempt to bias the mask set towards better accuracy for breakpoints by forcing the most compact mask to be in the set, and by limiting the width of the masks.

Similar search strategies are available that can efficiently find optimized mask set [13-15], although our search strategy specifically designed for assessing accuracy according to our tables. In any case, we know the accuracy table of the resulting optimized solution, and can judge whether it is adequate for indels.

The above global search strategy works well for sets consisting of a few masks, say *S* ≤ 10. For much larger sets of masks, the *S*-mask space becomes too large for effective sampling, and we instead prefer a iterative greedy search strategy. Here, the setup and techniques are similar to the global sampling algorithm, but we add masks one at a time to a growing mask set, each successive mask chosen by random sampling. The mask choice made at iteration *S* is the one that gives the greatest accuracy set at that point in the process, combined with the already chosen *S*-1 masks, and this is the sense in which it is a greedy search algorithm. Since the random sampling is focused on search for just one mask, the sampling is much more thorough, resulting in much better optimization of each individual mask. This process effectively generates mask sets of any total size, *S*, and moreover, because of the greedy addition process, using any subset of the first *s* < *S* masks from the ordered list would give a greedy optimum *s*-mask set, so smaller optimized sets can easily chosen. For example, in the BFAST distribution, we provide greedy sets containing *S*=40 masks, as well as the accuracy tables for using just the first *s*=1, *s*=2, *s*=3, …, so that one can conveniently choose a subset with any desired accuracy level, and which is furthermore already optimized relative to that accuracy specification. The greedy random search performs very similar to the global random search for smaller mask sets that method is suited for, so it seems that a greedy strategy is well suited to this problem.

#### Standard Optimized Mask Sets

The BFAST distribution includes greedy search optimized mask sets for reads from *L*=20 to *L*=100, and up to *S*=40 masks long, with associated accuracy tables for all choices of fewer masks *s* < *S*, so that it is convenient to choose a mask set for the read length and accuracy levels of interest for any specific alignment problem. More generally, the accuracy table calculation tools and random search tools described here are included in the distribution, and can be tailored to generate optimal masks sets for specific situations, for example a read technology where small indels are a dominant error mode, and thus should be weighted more prominently in the search process. Nonetheless, it is important to note that the full accuracy table of a mask set will always be known, so the performance specifications will be clear in any case

## Key size for the Human Genome

As noted in a previous section, choosing the index key size, *k*, involves a trade-off between the number of spurious CALs per read versus the accuracy of finding the genomic location of reads with variants. We consider this relationship in detail for the Human Genome, with the goal of picking a reasonable key size for index construction. In Table S1 and Table S2 we see the mean, minimum and maximum number of lookup locations, or CALs, that result per key when using an index of a given key size, *k*, as well as the fraction of keys that map uniquely and the contrasting fraction that map to many locations (>100), for a range of key sizes. In addition, both Figure S2 and Figure S3 show the distribution of the number of CALs per key in detail, for a range of key sizes. The specific data is for the special case of the mask that selects a contiguous *k*-mer as a key, but the results are similar for any mask of key size *k*. The results are obtained by indexing the Human Genome, forward and reverse complement, and then exhaustively looking up the genomic locations---the equivalent of CALs in this model calculation---of each of ~6·10^9^ location keys from this reference sequence, to obtain the total number per key. Note that the true location is included amongst the results, as all keys will have ≥ 1 CAL. As suggested by the theoretical estimates seen in previous sections, the percentage of keys that have just one CAL---thus mapping uniquely---dramatically increases from *k*=16 to *k*=18, and stabilizes around *k*=20. This strongly favors a key size of *k* =18-20, when that is not too large to compromise accuracy. For contrast, for a key size of 14, which encompasses the standard search key size of many alignment tools, the majority of keys still have between 10-100 CALs, implying a large overhead of spurious CALs that would result from using an index based on such a small key size.

However, we also see there is a substantial fraction of the Human Genome sequence that is highly repetitive, reflected by the roughly 20-40% of all keys that result in 10^2^-10^7^ CALs in the distributions shown in Figure S2 for *k* ≤ 16. These lie within repetitive sequence families that have correspondingly high copy number, as well as a high degree of homology within each family (> 90%). We observe that distribution of CALs becomes more unique when genome is encoded in ABI SOLiD color space. Referring to Table S1 and Table S2, we find that extending the key size up to *k*=50 is required to largely reduce these families uniquely indexed locations, and even then ~2% of 50-mers remain highly non-unique, with >100 CALs. Moreover, such a large key size (50 bases) cannot be used for accurate index sets for shorter reads, *L*=25-100, so reads from these repetitive locations will be impractical to lookup directly. Thus increasing key size beyond *k*=18-22 has little practical benefit for short read data. Instead, high CAL keys must simply be ignored in practice, and the alignment of repetitive sequence must be dealt with via means other than pure indexed lookup, such as paired end reads or longer-read technology.

Based on these considerations, we favor the key size range of *k*=18-22 for the Human Genome, although it may be necessary to go as low as *k*=14-16 to accurately align shorter reads in the *L*=25-30 base range.

## Index Set Selection

First, we survey the required number of indexes needed to achieve various desirable accuracy levels. We constructed optimized index sets using the global random sampling procedures outlined the previous sections, with key size *k*=18 and *k*=22 for read lengths of *L*=36 and *L*=50 respectively. The key size *k*=18 reflects the proper choice for the Human Genome given the shorter read length as noted above, while the read lengths are typical of the ones we have obtained in practice on our Illumina 1G, GAI and GAII sequencers. We increased the *k* to 22 for the 50bp reads as to further increase the uniqueness of each lookup, thus striving to remove false-positives. For each desired total number of indexes, we randomly sampled 50,000 possible mask sets and evaluated their accuracy, based on 50,000 random configurations from specific read variant categories---either reads with a given number of mismatches (0-10), or reads with a given size of insertion (0-20 base), or various combinations of the two. We retained the index set with the best accuracy for each such optimization effort, and we plot the various measures of accuracy versus number of indexes in the set in Figure S11, for read lengths of 36 and 50 respectively. We can see that for 36-base reads that a large number (>10) of indexes are required to be robust against reads with greater than 4 mismatches. In contrast, for 50-base reads, just 3 indexes can give a reasonable (>90%) accuracy. These results illustrate the value of longer read lengths for reducing alignment costs, measured as number of indexes required to achieve a desired level of accuracy.

In practice a large number of reads will have few or no variants, and contain few or no errors and therefore the overall accuracy in looking up CALs for a given read set will be an occurrence-weighted average of these individual accuracy measures, dominated by the modes with perfect or near-perfect lookup accuracy. Of course, if the performance level was judged insufficient for a particular application, a larger set of indexes, could be used to obtain any desired level of accuracy.

## Various Modes of BFAST

To demonstrate the ability of BFAST to trade-off speed for accuracy, we evaluated four modes of BFAST: *fast*, *moderate.speed*, *moderate.accuracy*, and *accurate*) (see Methods for BFAST settings). The four modes demonstrate BFAST’s ability to trade off increase sensitivity with progressively higher time requirements. The *fast* mode uses only one index and one offset, thereby performing only one lookup per read, which provides an indication of the raw lookup speed of BFAST. The *moderate.speed* mode also uses only one index, but all possible offsets in that index, thereby giving the fastest possible performance when fully utilizing one index type. The *moderate.accuracy* mode uses a strategy whereby there are two sets of indexes, with one index in the first set and three indexes in the second set, generating keys over all possible offsets. If no candidate alignment locations (CALs) for a read are found using the first set of indexes, the read subsequently searched using the second set of indexes. This can be a useful strategy in order to quickly find near-perfect matching reads using the first index set, and then spend more time searching for CALs for difficult, high-error or high-variant reads using the second index set.

The *accurate* mode uses an optimized set of 10 indexes, shown in Table S3-S6, and all possible offsets, in to maximize the likelihood that the correct alignment location will be contained within the set of CALs. To find such an optimized index set for BFAST *accurate,* we randomly search over all possible indexes to greedily add one index to the set. The initial index is a contiguous *k*-mer as its lookup key (*k=18* for reads less than 40bp, and *k=22* otherwise). Each additional index maximally improves the robustness to errors and variants. This method is incrementally performed until the index set reaches the desired mapping ability (see previous sections). A series of such indexes have been generated for the human genome and can be generated for any genome. This process permits the a priori assessment of sensitivity of a given index set for the likelihood of mapping reads types with preset error/variant distributions. The index sets can be optimized for the known target genome as well as the read length, or error/variant mode with their associated theoretical sensitivity statistics. A detailed description is provided in Supplemental Tables S3-S14. For demonstration here, BFAST *accurate* is performed with 10 indexes, but larger index sets and strategies could be applied by the user. This index search strategy produces sets of indexes with high accuracy compared to manually designing the index set, although other search methods for finding sets of such indexes are available [13-15]. When comparing the various modes of BFAST, we required that the chosen alignment for a read was at least two mismatches away from the next best alignment. This reduces our sensitivity but more importantly decreases our false-mapping rate.

We examine in Figure S14 the effect of errors alone when mapping reads for 36 base-pair reads and 50 base-pair reads to the human genome by various modes of BFAST using simulated datasets (see Methods). As expected, BFAST running in *accurate* mode, which uses a larger number of indexes of the human genome, was able to map correctly the largest fraction of the simulated reads, and is resistant to a larger number of errors. For instance, for 36 base-pair reads only the BFAST *accurate* was able to map 79.9%, 77.3%, 72.8%, and 60.2% for 0-3 errors (Figure S14A) and dropped to 27.7% mapping for a 36 base-pair read with 4 errors (a 11% error rate). For 50 base-pair reads, the BFAST *moderate.accuracy* was able to map 90% of all reads with up to 4 mismatches. BFAST *accurate* was able to map 87.6%, 85.4%, 84.6%, 82.8%, 80.5%, and 77.4% of reads with 0-5 mismatches, which in the 5 mismatch case is equivalent to tolerating a 10% error rate in the quality of the sequence and still detecting the accurate placement (Figure S14C). To increase the sensitivity we could apply a more inclusive post-alignment filter. Additionally, we trade off in sensitivity in BFAST when we use more than one index (*moderate.accuracy* and *accurate* modes) for 50 base-pair reads, results mostly in increased search time. However, repetitive locations in the genome may generate hundreds if not thousands of CALs. Therefore, for practical purposes, we typically set an upper limit on the number CALs for the human genome, for example 1028 for the *accurate* mode. This leads to an inflation of false-negatives as the correct location was identified by could not be accurately ascertained from many equally good locations. This is predominantly the issue for the observed moderate decrease in sensitivity with up to 5 mismatches that makes the true location undetermined (Figure S14C). This could be mitigated by using indexes with longer key-sizes to possibly procure unique CALs for each look-up in an index or by selecting for informative keys (see Supplementary Materials), but at the expense of additional lookups. This is an active area of interest in that the correct placement of reads even from repetitive sequences will have an impact on the ability to identify structural variants from paired end data. Additionally, of those reads mapped, we observe a lower false positive mapping rate when searching with greater sensitivity in the more accurate modes (Figure S14B and S14D). In general, the BFAST *accurate* setting is the preferred mode for genomic sequencing applications with highly complex genomes and the desire to identify base differences, as well as short insertions and deletions. While there are likely to be uses of the more rapid mapping approaches or specialized index strategies, here we focus on the BFAST *accurate* mode based on 10 indexes only to demonstrate areas of improved alignment performance made possible by BFAST.

## BFAST timing breakdown

In order to consider future areas to improve performance characteristics, it is important to analyze on what part of the alignment process BFAST spends its time. In Figure 5, the timing for each of the four real-world datasets is divided into the time spent finding CALs versus that used to perform local alignments for each of the four chosen BFAST modes. Both processes are highly dependent on each other. For example, if more CALs are found in the first step, more local alignments need to be performed, which can be seen in all four datasets examining specifically the BFAST *accurate* mode. This increase in CALs returned can be mediated by using a longer key-size or ignoring reads that return too many CALs prescribed by some upper limit (see Supplementary Materials for more complete discussion on parameter setting).

For the Illumina datasets, the settings used in BFAST, including setting maximum upper limits on the number of CALs returned per lookup *K* and total number of CALs aggregated *M* (see Supplementary Materials) have balanced the CAL lookup step and the local alignment step for 50bp reads.

For the ABI SOLiD datasets, BFAST spends the majority of its time on the local alignment step. This local alignment has a fundamental limit on speed and can be only reduced by a constant factor based on the number of candidate alignment locations. Also, the local alignment for ABI SOLiD color space requires BFAST to simultaneously decode and align the read, thereby increasing the search space in the local alignment step, translating into a longer running time [17]. However, identifying CALs in color space is very efficient since the same CAL search algorithm is performed on an encoded color space reference. Nevertheless, we investigate ABI SOLiD local alignment performance in the Supplementary Materials where we consider only mismatches and encoding errors, observing that a significant performance increase can be had by reducing the dimensionality of the problem at the cost of ignoring possibly relevant biological events (indels).

## Algorithm Settings for Simulations

### BFAST

We examined four different modes of BFAST to demonstrate the flexibility in terms of speed and accuracy. The four BFAST modes were *fast*, *moderate.speed*, *moderate.accuracy*, and *accurate*, listed in order of increasing accuracy at the cost of speed. We utilized BFAST version 0.5.2.

The index sets for aligning the Illumina 36 and 50 base pair reads to the Human Genome can found in Table S3 and Table S4 respectively. The index sets for aligning the ABI SOLiD 25 and 50 base pair reads to the human genome can be found in Table S5 and Table S6 respectively.

Additionally, for the *fast* BFAST mode, we choose to only examine the first offset of each index, thereby producing one key per read per index. For the other BFAST modes *moderate.speed*, *moderate.accuracy*, and *accurate* all possible offsets were used.

For all BFAST modes, we used a minimum alignment score of zero to help filter poor quality alignments, recognizing this as a very inclusive filter. Using Figure S4 as a guide to obtain an approximate 90% inclusion rate of the correct CAL in the CAL set, we chose the upper limits on the number CALs per key *K* and the number of CALs per read *M*. Since the results shown in Figure S4 are for one index, we scaled accordingly for the number of indexes used in each of the four modes. For the BFAST *fast* mode we used the options -K 8 -M 8, for the BFAST *moderate.speed* mode we used the options -K 8 -M 128, for the BFAST *moderate.accuracy* mode we used the options -K 8 -M 384, and for the BFAST *accurate* mode we used the options -K 8 -M 1280. We required that the chosen alignment uniquely had the best alignment score for the inclusive (I) post-alignment filter. For the exclusive post-alignment filter, we required that the chosen alignment effectively was at least two mismatches closer to the reference than the next best alignment. To assess the effective distance between alignments, we used the difference between alignment scores normalized by the difference between a match and mismatch.

### BLAT

We used the BLAT stand-alone program version 34. We evaluated various input settings and found that the following command line options gave the best results in terms of performance and accuracy: standard.15 -out=maf -noHead -t=dna -q=dna -maxIntron=3 -minScore=30 -minIdentity=85 -stepSize=5 -tileSize=15. Due to memory constraints, we evaluated each chromosome separately. Due to timing constraints, for both the Illumina 36bp and 55bp dataset, we selected the first 1 million reads and extrapolated the timing and mapping results for the entire dataset.

### Bowtie

We used bowtie version 0.10.0 using the default settings. We tried the -n 3 option but surprisingly this resulted in less sensitive alignments with lower accuracy (results not shown). We also set -l 32 and filtered all alignments that had a mapping quality less than 10.

### BWA

We used BWA version 0.4.9 using the default settings. To allow for a longer gap extension, we set -e 10. We found that -l 36 also produced fast and accurate results. To allow for sensitive alignments, we set the -n 3 option and filtered all alignments that had a mapping quality less than 10.

### MAQ

We used MAQ version 0.7.1 using the default settings. For aligning to nucleotide space data, we used the -e 1000000 command line options. For aligning to ABI SOLiD color space, we used the -c -e 100000 command line options. For MAQ we applied a minimum mapping quality of 1 and 10 for the inclusive (I) and restrictive (R) post-alignment filters.

### SHRiMP

We used SHRiMP version 1.1.0, utilizing settings suggested by the author for the various read lengths used in our simulated and real-world datasets. The command line options suggested were -P -s 1111111001111111 -n 3 -B with the additional option - r R where R matches the read length in the given dataset. Due to timing constraints, for the Illumina 36bp and 55bp datasest, we selected the first 1 million reads, extrapolating the timing and mapping results for both datasets.

### SOAP

We used SOAP version 1.011, using the *soap.huge* version for execution with large amounts of memory. We evaluated various input settings and found that the following command line options gave the best results in terms of performance and speed: -s 12 -r 2.

## Implementation

### Memory Requirements

In the BFAST implementation, to store each index---which contains in aggregate all positions in the genome---we use one byte to encode the chromosome and four bytes to encode each position within a chromosome. This could be further compressed to four bytes total (2^32^ positions) for indexing the Human Genome, but for simplicity we choose the former implementation. For the Human Genome, we are able to store an index in approximately 17GB of memory, including the reference genome sequence itself as well. It is interesting to note that amount of memory required to store both the index and the reference genome is simply a function of the reference genome size but neither a function of the read length nor any other parameter. Therefore we can re-use indexes across multiple sequence read data sets that have a common reference genome.

The size of the hash table for an index is a function of the hash key size, *j*.

In practice we store the 4*^j^* different key entries using one 32-bit unsigned integers.

To look-up a value---consisting of byte start and stop locations in the primary index---we simply convert the *j*-length string to a base four integer and index directly into the hash table using this integer as the pointer. We store only the start positions, inferring the stop position from the subsequent hash entry. In detail, instead of storing start and end positions in the main index, we can fill empty start positions by the next defined hash entry. In this case, all start positions will be filled. To infer if a start position is defined, or has just been filled, we observe that a start position is defined only if the next filled or defined start position has a different value than the current start position (in this case it must be greater than). We observe that the last few entries in the hash table can be filled or defined, and this corner case is easily handled by inserting a value greater than the length of the main index in the filled case as to differentiate the filled value from the corner case. The storage size of the hash is thus 4 bytes times the 4^j^. For example, we are able to store a hash width *j*=14 in 1 GB of memory.

Thus, in total, the complete memory requirement for a single Human Genome index---including the positional data, the reference sequence, and the *j*=14-mer hash table---is ~17 GB. Because of this, when working with the Human Genome, a shared memory system with ~24 GB of dynamic memory provides optimal performance, allowing several GB for program and operating system overhead. However, if less memory is available, BFAST can separate the indexes into pieces to accommodate the memory limit, although this will proportionately increase the run time, as all reads

will be processed in full through each segment of the index, albeit it with a greater miss rate for the lookup.

#### Program Organization

BFAST is broken up into four sequential command-line programs. The first program *bpreprocess* pre-computes the indexes and their hash tables according to their desired layouts. The second program *bmatches* takes as input the reads as well as the indexes and outputs a list of CALs. The third program *balign* aligns separately each CAL using the well-known Dynamic Programming optimal sequence alignment algorithm.

The fourth program *bpostprocess* is used to help choose the local alignment based on various criteria. Other utilities, including but not limited to utilities that search for optimal mask sets, generate and evaluate simulated data, compute empirical paired distance, and produce error rate estimations, can be found in a special *butil* folder. Additionally, BFAST is able to produce output in the SAM format (<http://samtools.sourceforge.net/>).

The first three main programs are multi-threaded to enable faster computation and BFAST is implemented for Mac OS X and any Linux/Unix system utilizing the POSIX thread interface. Additionally, we utilize the GNU Autotools to automate compilation and installation. To facilitate the use of our program on a cluster, we allow the user to specify a subset of the reads to be aligned, which then can be merged after each node in the cluster has completed its respective alignment. Complete documentation is available in the form of a reference manual, which is provided with the source code, all of which are freely available for non-profit academic use.

#### Multi-threading Performance

Using the POSIX thread interface, we are able to spawn multiple threads both during the CAL search step and the local alignment step.

In Figure S9 we present the empirical performance of performing 10 million CAL lookups when a various number of threads are used in parallel for both nucleotide space and ABI SOLiD color space data. We aligned the reads to the Human Genome in the *fast* BFAST mode for 50bp reads.

In Figure S10 we present the throughput of local alignments for various number of threads used in parallel. We performed two different local alignment algorithms. The first performed full alignment (*FA*), which considered mismatches, insertions, deletions, and in the case of ABI SOLiD color space data, considered encoding errors. Next we performed a mismatch only alignment (*MM*), which considered only mismatches, and in the case of ABI SOLiD color space data, considered encoding errors. We performed 25 million local alignments for all cases except the full alignment in color space, where we performed only 1 million local alignments due to time constraints. In all cases, we aligned the 50bp reads with each reference spanning 70bp.

For both these evaluations, we used a dual quad-core Intel Xeon E5420 machine at 2.5GHz, with 32GB of RAM and 2TB of RAID 0 disk space.

It is interesting that we do not see performance scale as expected, except for local alignments performed in color space, where the local alignment is more CPU intensive.

This may be due to the memory bandwidth upper limit, operating system process scheduler overhead, and disk performance. Since the indexes may be very large for the Human Genome (>16GB), it is very unlikely that a lookup in the index or the reference genome (for Humans) will hit in the cache, and therefore must be retrieved from RAM or memory. Therefore spawning too many threads may saturate the memory bus and therefore cause degraded performance due to waiting on RAM requests and context switching.

## Illumina Datasets

For demonstration purposes, we used a 10.9 million 36 base read data set from the human genome. In total, 33 different regions with known mutations across 5 genes were PCR amplified individually and pooled. Amplicon sizes ranged from 191bp to 762bp. After purifying each amplicon with QIAGEN PCR Purification Kit, the amplicons were pooled in one tube to create an equal mixture of all products. The sequencing library from the genomic fragments was prepared using manufacturer provided genomic library preparation protocol version 2.3 (Illumina, La Jolla, Ca). Specifically, this dataset consisted of sequence from PCR products known to contain 13 mismatches, as well as 6 small insertions and 6 small deletions, and sequenced at a depth of coverage generally exceeding 1000-fold.

We also analyze here 3.5 million 55 base paired-end reads of human genomic sequence from our Illumina GAII sequencer. Libraries were generated from genomic DNA. We then selected only the first end of the pair, giving us 3.5 million 55 base pair single-end reads for alignment.

## ABI SOLiD Datasets

One million reads from two different runs of in house generated ABI SOLiD sequencer data was used for all comparisons, as this is a sufficient dataset to offer reasonable comparison. Both datasets consisted of sequences from human genomic DNA, generated by using standard 25 base and 50 base manufacturer supplied protocols.

# Figure Legends

## Figure S1 Legend

**The log_10_ number of spurious candidate alignment locations (CALs) for varying key sizes according to Equation S1**. The plot is for a four letter DNA alphabet, and read length *L*=50. The four lines correspond to four different genome sizes, *G*. The expected number of spurious CALs for the Human Genome for *k*=11 is approximately 60,000, whereas for *k*=18 this number is 3.

## Figure S2 Legend

**Distribution of short sequence CALs to the Human Genome at various *k*-mer keys.** For varying key sizes, *k*, the number of lookup locations for each *k*-mer key from the Human reference genome was calculated, forward and reverse complementary sequence included (~6·10^9^ keys). The figures show the computed percentage of keys that have a given number of genomic locations, the equivalent of CALs for index lookup. For the non-repetitive part of the genome, a key size of *k*=18-22 is adequate for essentially unique lookup, except for the repetitive portion of the genome, indicated by the large tail populations in the histograms with 10^2^-10^7^ locations. The most highly repetitive keys are in the conserved portion of the *Alu* repeat family.

## Figure S3 Legend

**Distribution of short sequence CALs to the Human Genome at various *k*-mer keys in ABI SOLiD color space**. Similar to Figure S2, the number of lookup locations under varying parameters are plotted, but instead for the Human Genome when encoded using ABI SOLiD color space.

## Figure S4 Legend

**Performance of imposing upper limits on the number of CALs returned by a lookup and the number of CALS compiled for a read.** Using simulated 36 bp reads from the Human Genome, and an 18 base contiguous mask looking up over all possible starting locations in the read, the performance of using various values of parameters *K* and *M* is plotted. The fraction of the time the correct CAL is included in the CAL set for various values of *K* (the maximum number of CALs per key) and *M* (the maximum number of CALs per read) is plotted. We see that the value of *M* has a dramatic effect on our performance regardless of *K*, whereas given *M* various values of *K* can help maximize the performance. Limiting the number of CALs in total will dramatically affect the running time due to the decreased number of local alignments required.

## Figure S5 Legend

**Accuracy results of alignment algorithms from simulated 36 base reads with a given length deletion and errors.** We present an assessment of the accuracy of four modes of BFAST and various other widely used algorithms based on aligning simulated data sets of with reads of length 36 bases with a given a length deletion and ranging over various numbers of errors modeled as mismatches (see Methods). We see that the difficulty of aligning deletions is caused by errors

## Figure S6 Legend

**Accuracy results of alignment algorithms from simulated 36 base reads with a given length insertion and errors**. We present an assessment of the accuracy of four modes of BFAST and various other widely used algorithms based on aligning simulated data sets of with reads of length 36 bases with a given a length insertion and ranging over various numbers of errors modeled as mismatches (see Methods). We see that the difficulty of aligning insertions is caused by errors

## Figure S7 Legend

**Accuracy results of alignment algorithms from simulated 50 base reads with a given length deletion and errors.** We present an assessment of the accuracy of four modes of BFAST and various other widely used algorithms based on aligning simulated data sets of with reads of length 50 bases with a given a length deletion and ranging over various numbers of errors modeled as mismatches (see Methods). We see that the difficulty of aligning deletions is caused by errors

## Figure S8 Legend

**Accuracy results of alignment algorithms from simulated 50 base reads with a given length insertion and errors**. We present an assessment of the accuracy of four modes of BFAST and various other widely used algorithms based on aligning simulated data sets of with reads of length 50 bases with a given a length insertion and ranging over various numbers of errors modeled as mismatches (see Methods). We see that the difficulty of aligning insertions is caused by errors.

## Figure S9 Legend

**The time to find CALs for various number of threads used in parallel.** We present the empirical performance when a various number of threads are used in parallel for both nucleotide space and ABI SOLiD color space data.

## Figure S10 Legend

**The throughput of local alignments for various number of threads used in parallel.**

We present the empirical performance when a various number of threads are used in parallel for both nucleotide space and ABI SOLiD color space data. We performed two different local alignment algorithms. The first performed full alignment (*FA*), which considered mismatches, insertions, deletions, and in the case of ABI SOLiD color space data, considered encoding errors. Next we performed a mismatch only alignment (*MM*), which considered only mismatches, and in the case of ABI SOLiD color space data, considered encoding errors.

## Figure S11 Legend

**The effect of larger index sets versus various measures of accuracy.** For the lookup of specific classes of variant reads, we plot the accuracy results for various index sets. A and C show the effect of the number of indexes on accuracy, for optimized mask sets for 36-base reads (A) and 50-base reads (C), with accuracy measured against the number of mismatches in the read relative to the reference. B and D show the effect of the number of indexes on accuracy when considering 36-base reads and 50-base reads with a contiguous 1 base and 5 base insertion respectively combined with 0--6 read errors (as mismatches). Utilizing more indexes can increase the overall accuracy, although the power to map the reads becomes reduces as the number of errors increase

## Figure S12 Legend

**Comparison of time spent by BFAST finding candidate alignment locations (CALs) versus time spent by BFAST on local alignment.** For the four real-world datasets and four demonstrated modes of BFAST (see Methods), the time for performing each mode of BFAST is recorded on the y-axis and each of the four datasets. Time spent on finding CALs is referred to as ‘matching’ (M) and time spent on local alignment is referred to as LA. Color coding of graphs for the BFAST modes are indicated in the inset of the final panel. The balanced timing for the Illumina datasets can be attributed to selecting parameters *K*, the upper limit of CALs allowed to be returned by a lookup, and *M*, the maximum number of CALs aggregated for a read before ignoring the read (see Supplementary Materials). Due to additional need to account for errors in the encoding in ABI SOLiD data, the majority of the time was spent performing local alignment for this type of data.

## Figure S13 Legend

**The effect of post-alignment filters on the sensitivity and accuracy of the BFAST *accurate* mode**. We present an assessment of the sensitivity and accuracy of the BFAST *accurate* with various post-alignment filters applied. The assessment is based on aligning simulated data sets of with reads of length 50 bases with a given a length insertion and ranging over various numbers of errors modeled as mismatches (see Methods). We pick the unique best scoring alignment in each case, varying the requirement that the next best alignment must be effectively 0-5 mismatches farther from the reference than the chosen alignment. We see the trade-off between sensitivity and accuracy as more stringent filters are applied.

## Figure S14 Legend

**Evaluation of BFAST alignment modes from simulated 36 and 50 base pair reads from the Human Genome**. Four modes of BFAST were compared based on their sensitivity (percent of all reads that were mapped correctly) and accuracy (percent of mapped reads that were mapped correctly), which is plotted as the y-axis of each plot. Sequence lengths of 36 (A and B) and 50 (C and D) bases were considered with a given number of errors indicated along the x-axis of each plot. Errors were modeled as mismatches drawn from the human genome. The alignment method is indicated as colored lines per the key within the first figure panel. Sensitivity is shown in panels A and C, while accuracy is shown in panels B and D.

# Figures

## Figure S1


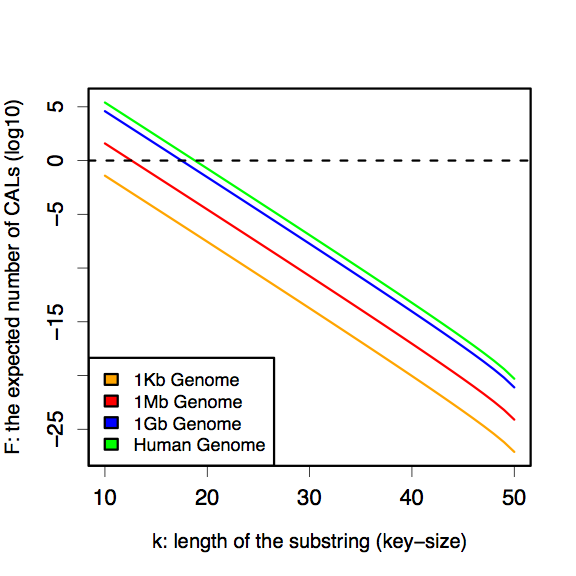


## Figure S2


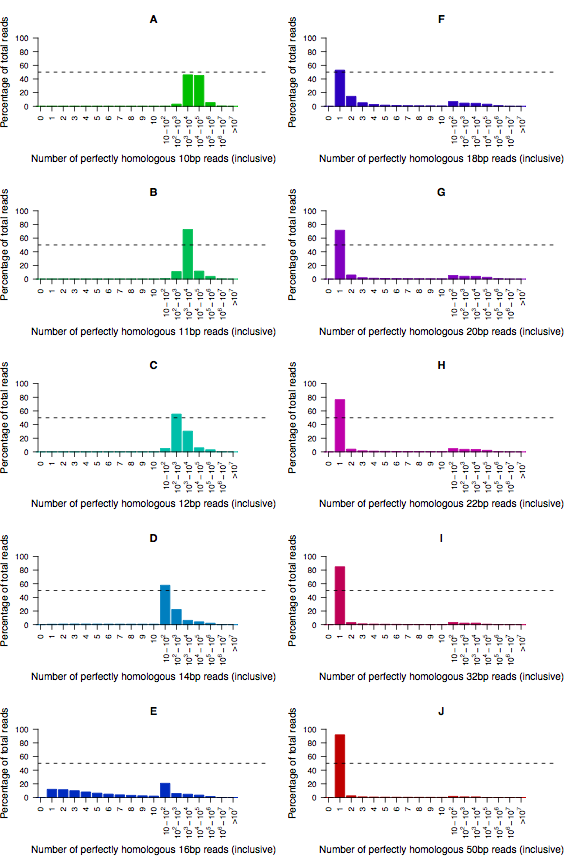


## Figure S3


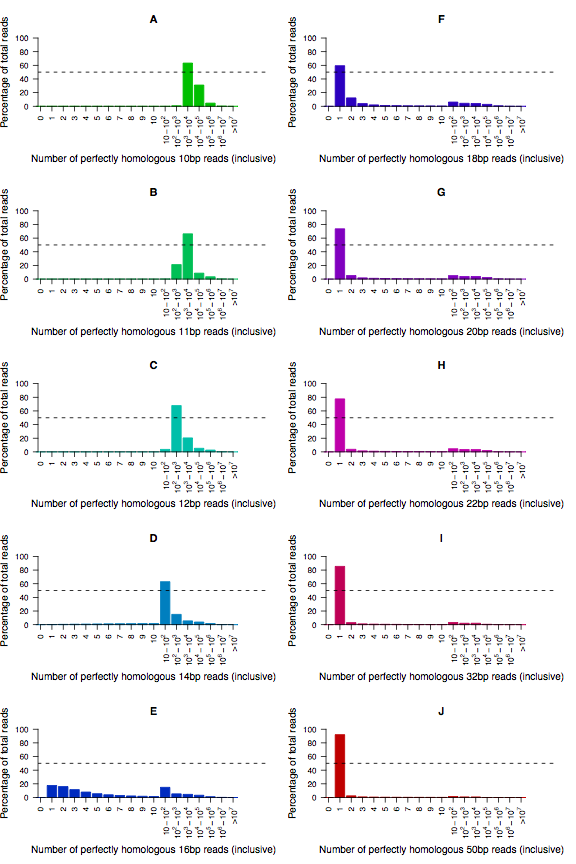


## Figure S4


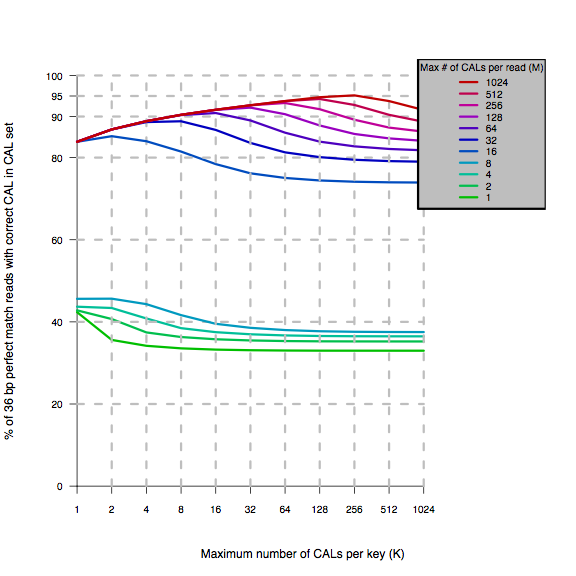


## Figure S5


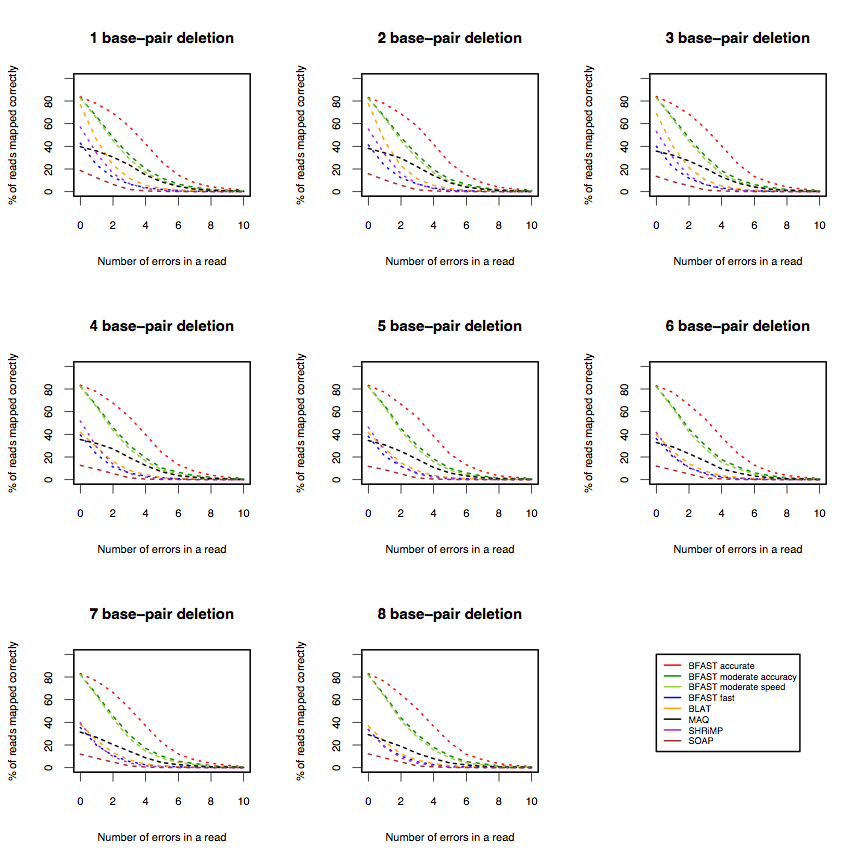


## Figure S6


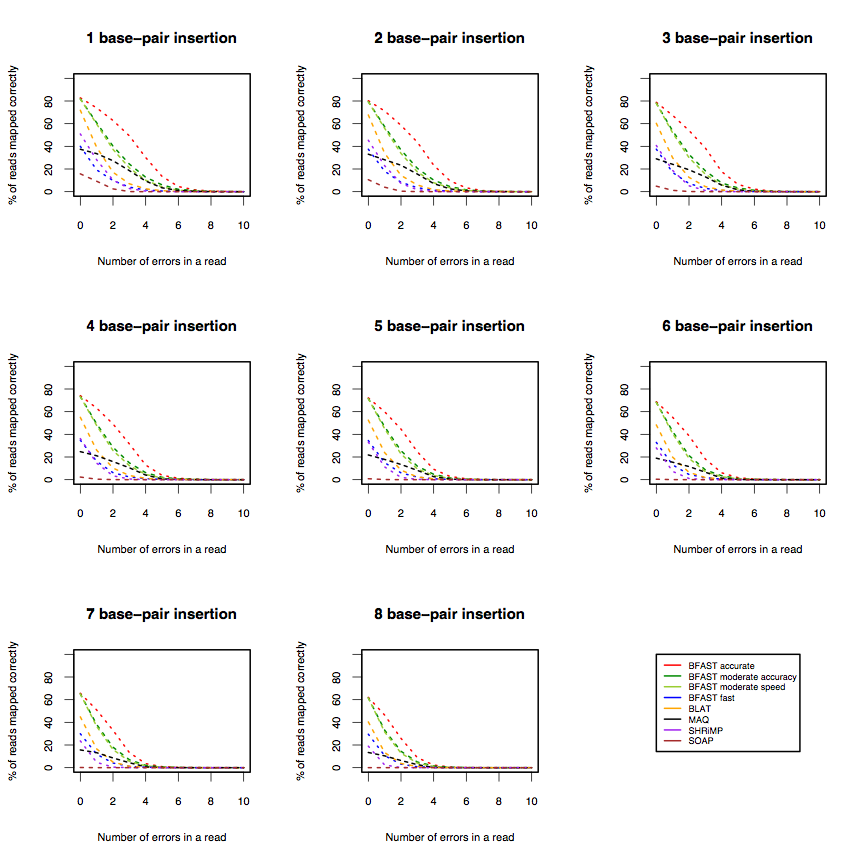


## Figure S7


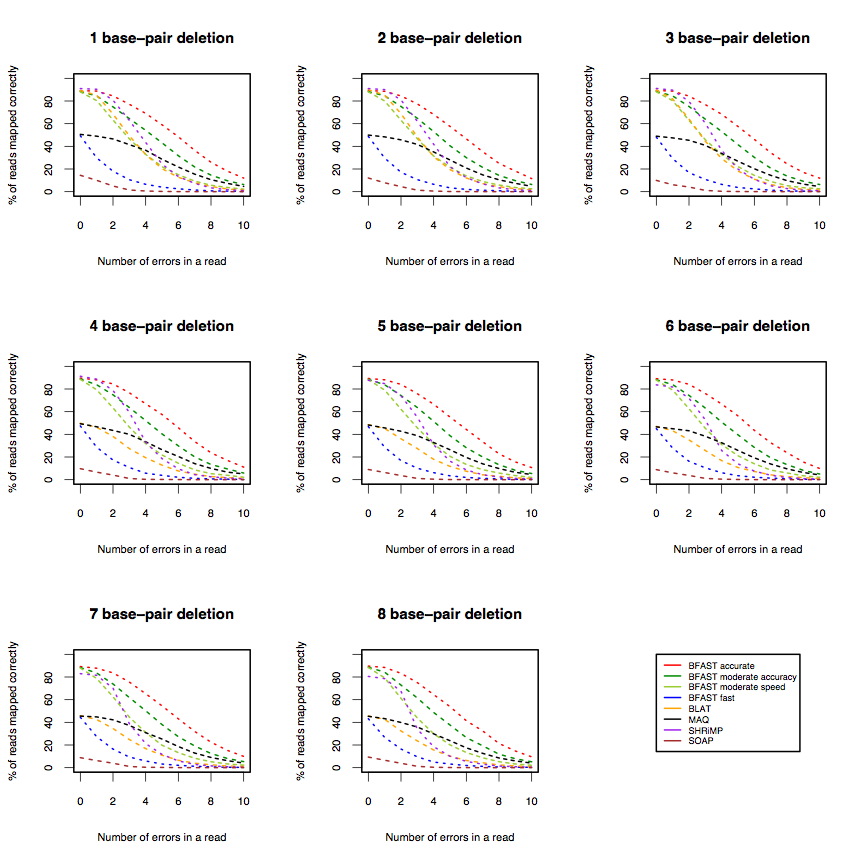


## Figure S8


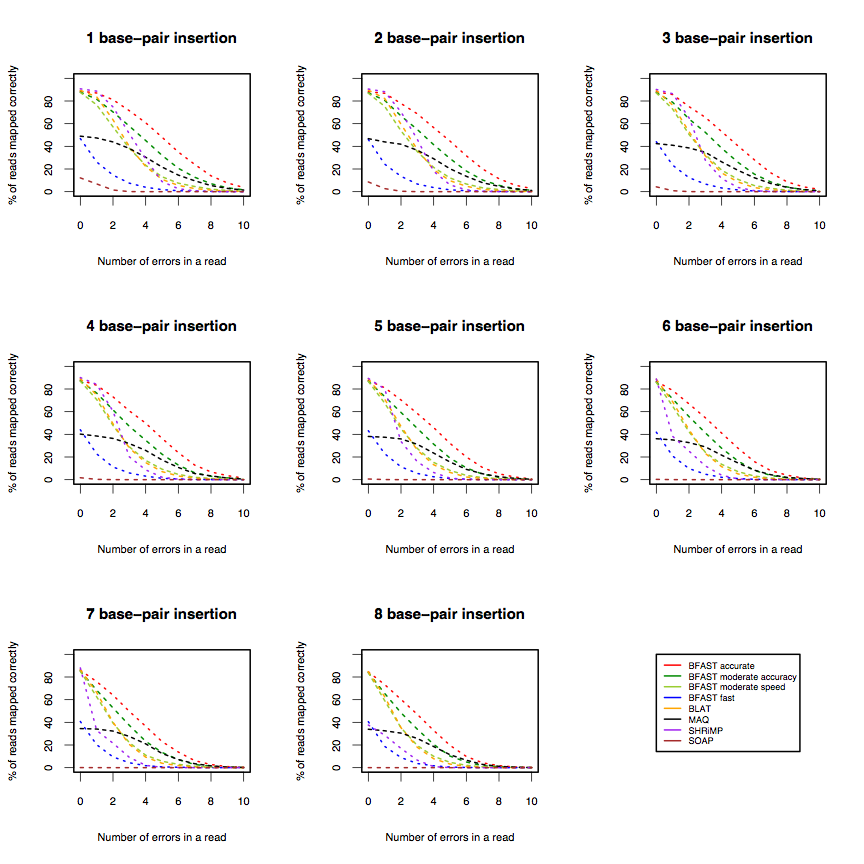


## Figure S9


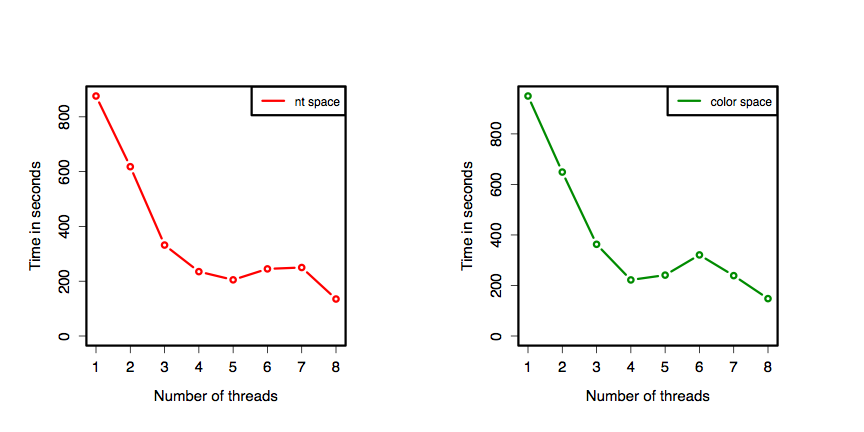


## Figure S10


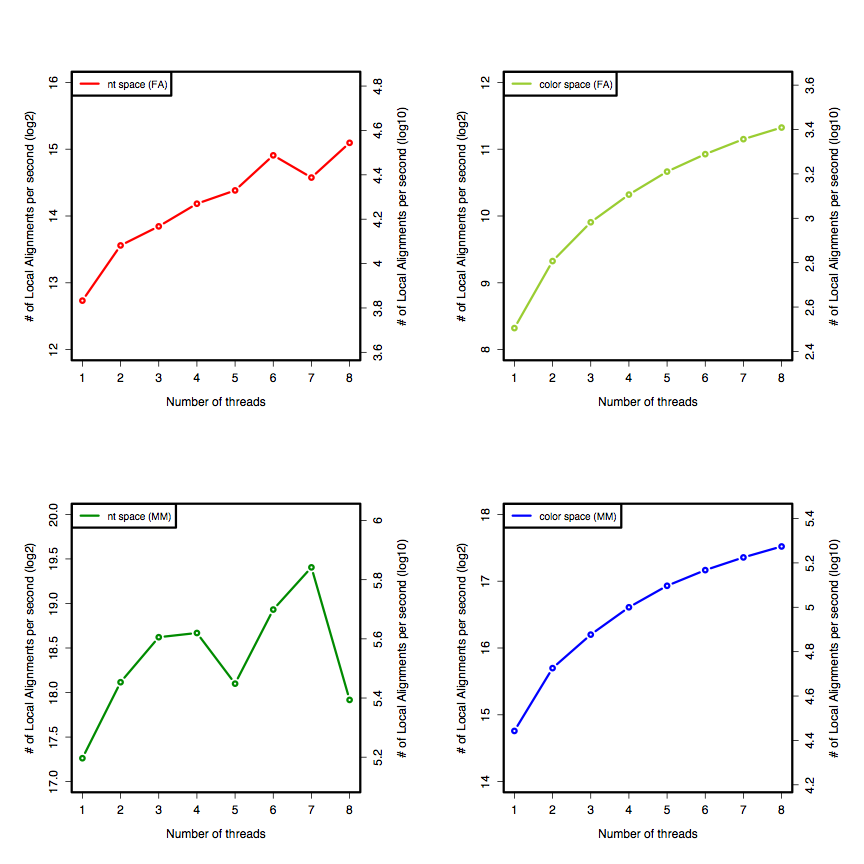


## Figure S11


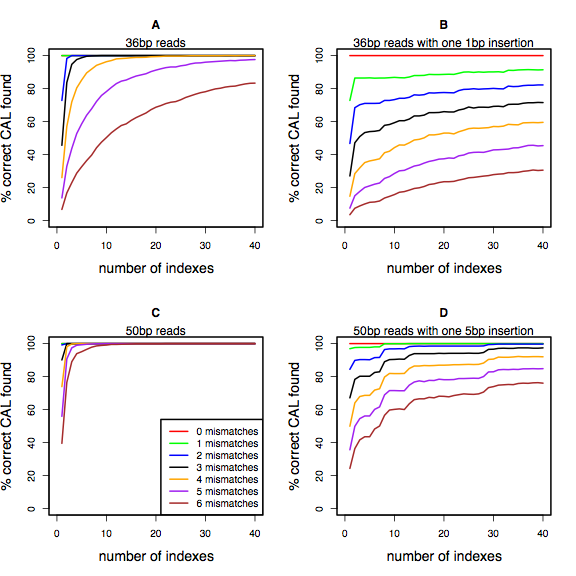


## Figure S12


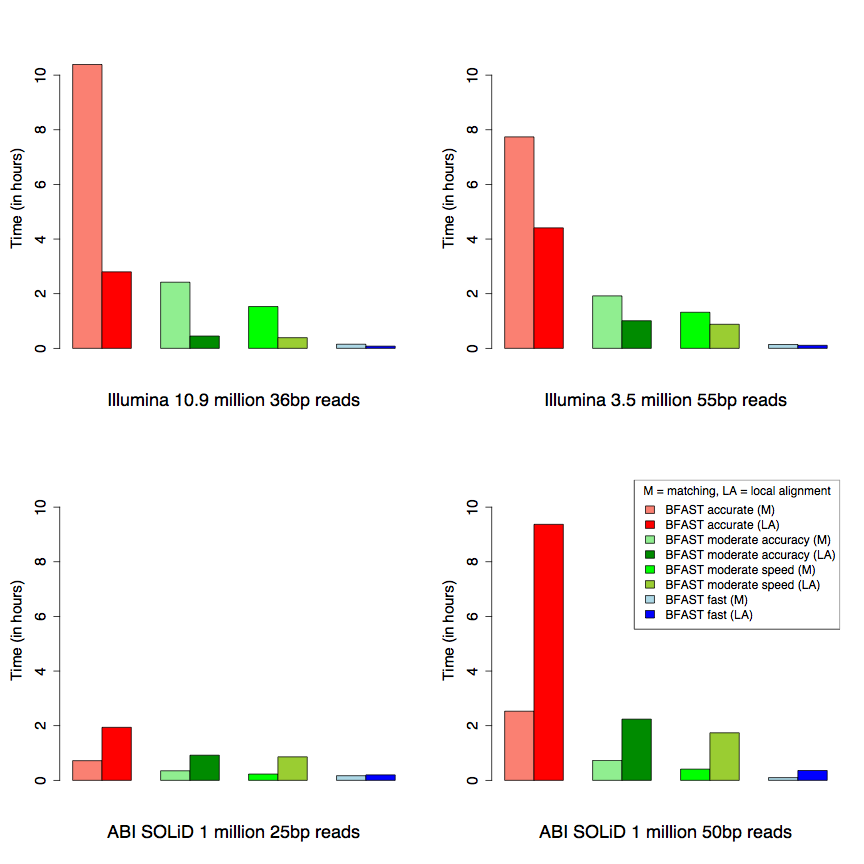


## Figure S13


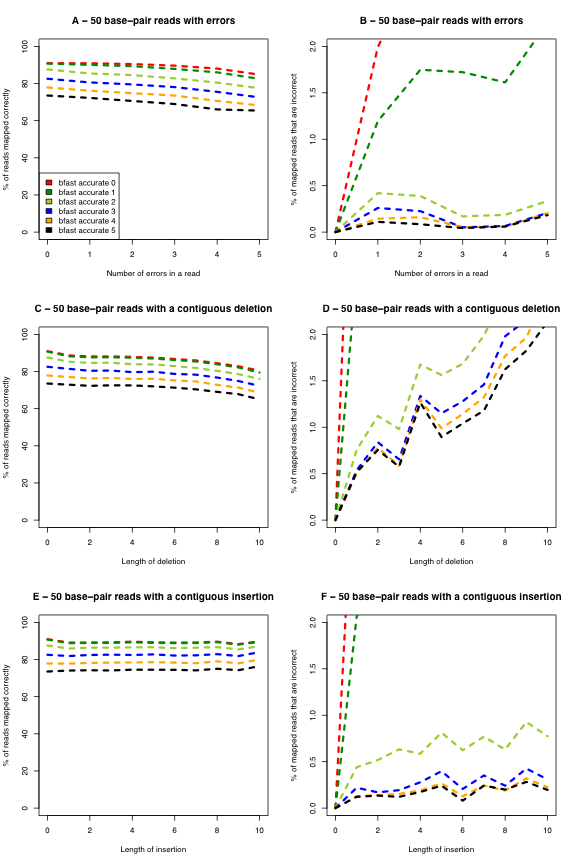


## Figure S14


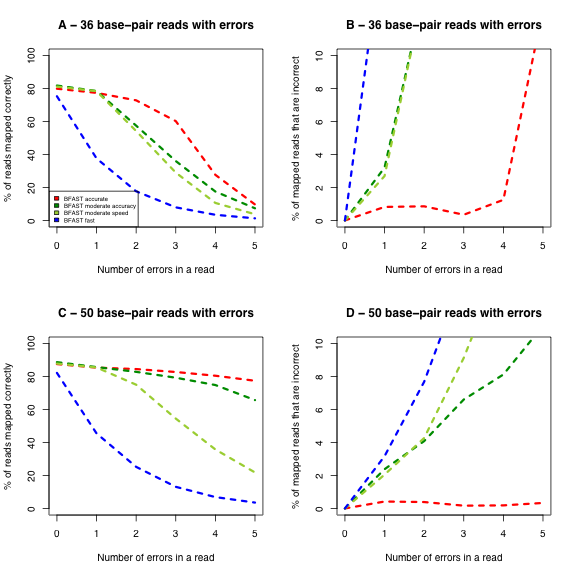


# Tables

## Table S1

| Key size (k) | Mean | Min | Max | % Unique Reads | % with ≤ 100 CALs |
| --- | --- | --- | --- | --- | --- |
| 10 | 5451.25 | 5 | 5928152 | 0 | 0.04 |
| 11 | 1362.85 | 1 | 4925231 | 0 | 0.57 |
| 12 | 341.63 | 1 | 4127159 | 0 | 5.11 |
| 14 | 25.14 | 1 | 2878063 | 0.54 | 65.11 |
| 16 | 3.58 | 1 | 1974053 | 12.13 | 84.19 |
| 18 | 1.56 | 1 | 1340823 | 53.14 | 87.14 |
| 20 | 1.31 | 1 | 897954 | 71.68 | 88.91 |
| 22 | 1.25 | 1 | 584002 | 76.7 | 90.34 |
| 32 | 1.14 | 1 | 160995 | 84.99 | 95.01 |
| 50 | 1.07 | 1 | 16905 | 91.88 | 98.34 |

**Statistics for the number of lookup locations for varying key sizes.** For varying key-

sizes, *k*, we calculated the number of lookup locations for each k-mer key from the Human reference genome, forward and reverse complementary sequence included (∼6·10^9^ keys). This table shows the minimum, mean, and maximum number of lookup locations (equivalent to CALs for an index lookup) based on data for all the k-mer keys from the reference. The table also summarizes the percentage of total keys that were unique (i.e. having exactly one CAL) and the percentage of keys that were at least not highly repetitive, defined as having less than 100 CALs. The data shows that there is a unique part of the genome that is well indexed by a key size of ∼18, plus a repetitive ∼10% of the genome that requires on the order of a 50-mer key for unique lookup.

## Table S2

| Key size (k) | Mean | Min | Max | % Unique Reads | % with ≤ 100 CALs |
| --- | --- | --- | --- | --- | --- |
| 10 | 5451.25 | 29 | 9879690 | 0 | 0 |
| 11 | 1362.81 | 2 | 8272368 | 0 | 0.16 |
| 12 | 340.71 | 1 | 6919222 | 0 | 3.7 |
| 14 | 22.13 | 1 | 4780644 | 0.23 | 73.32 |
| 16 | 2.83 | 1 | 3258810 | 17.82 | 85.47 |
| 18 | 1.46 | 1 | 2202282 | 59.64 | 87.93 |
| 20 | 1.28 | 1 | 1791878 | 73.87 | 89.57 |
| 22 | 1.23 | 1 | 1524054 | 77.82 | 90.91 |
| 32 | 1.14 | 1 | 561736 | 85.52 | 95.31 |
| 50 | 1.06 | 1 | 32258 | 92.11 | 98.42 |

**Statistics for the number of lookup locations for varying key sizes.** For varying key-

sizes, *k*, we calculated the number of lookup locations for each k-mer key from the Human reference genome, forward and reverse complementary sequence included (∼6·10^9^ keys) when the genome is encoded in ABI SOLiD color space. This table shows the minimum, mean, and maximum number of lookup locations (equivalent to CALs for an index lookup) based on data for all the k-mer keys from the reference. The table also summarizes the percentage of total keys that were unique (i.e. having exactly one CAL) and the percentage of keys that were at least not highly repetitive, which we deﬁne as having less than 100 CALs. The data shows that there is a unique part of the genome that is well indexed by a key size of 18, plus a repetitive ∼10% of the genome that requires on the order of a 50-mer key for unique lookup.

## Table S3

| Mask | Key size (k) and key width (w) | 36bp reads | | | |
| --- | --- | --- | --- | --- | --- |
|  |  | 1 | 2 | 3 | 4 |
| M1 = 111111111111111111 | (k=18, w=18) | M | M | M | M |
| M2 = 11110100110111101010101111 | (k=18, w=26) | M | S |  |  |
| M3 = 11111111111111001111 | (k=18, w=20) | M | S |  |  |
| M4 = 1111011101100101001111111 | (k=18, w=25) | M | S |  |  |
| M5 = 11110111000101010000010101110111 | (k=18, w=32) | M |  |  |  |
| M6 = 1011001101011110100110010010111 | (k=18, w=31) | M |  |  |  |
| M7 = 1110110010100001000101100111001111 | (k=18, w=34) | M |  |  |  |
| M8 = 1111011111111111111 | (k=18, w=19) | M |  |  |  |
| M9 = 11011111100010110111101101 | (k=18, w=26) | M |  |  |  |
| M10 = 111010001110001110100011011111 | (k=18, w=30) | M |  |  |  |

**Index sets used by the four modes of BFAST for 36 base pair Illumina reads from Human**

**Genome.** The masks sets for each of the four BFAST modes: *accurate*, *moderate*.*accuracy*, *moderate speed*, and *fast* are listed under the columns 1, 2, 3, and 4 respectively optimized for 36 base pair reads from the Human Genome. The masks used in the main indexes are listed as M, and the masks used in the secondary indexes are listed as S. The key size and key width are indicated next to each mask.

## Table S4

| Mask | Key size (k) and key width (w) | 50bp reads | | | |
| --- | --- | --- | --- | --- | --- |
|  |  | 1 | 2 | 3 | 4 |
| M1 = 111111111111111111 | (k=22, w=22) | M | M | M | M |
| M2 = 1111101110111010100101011011111 | (k=22, w=31) | M | S |  |  |
| M3 = 1011110101101001011000011010001111111 | (k=22, w=37) | M | S |  |  |
| M4 = 10111001101001100100111101010001011111 | (k=22, w=38) | M | S |  |  |
| M5 = 11111011011101111011111111 | (k=22, w=26) | M |  |  |  |
| M6 = 111111100101001000101111101110111 | (k=22, w=33) | M |  |  |  |
| M7 = 11110101110010100010101101010111111 | (k=22, w=35) | M |  |  |  |
| M8 = 111101101011011001100000101101001011101 | (k=22, w=39) | M |  |  |  |
| M9 = 1111011010001000110101100101100110100111 | (k=22, w=40) | M |  |  |  |
| M10 = 1111010010110110101110010110111011 | (k=22, w=34) | M |  |  |  |

**Index sets used by the four modes of BFAST for 50 base pair Illumina reads from Human Genome.** The masks sets for each of the four BFAST modes: *accurate*, *moderate*.*accuracy*, *moderate speed*, and *fast* are listed under the columns 1, 2, 3, and 4 respectively optimized for 50 base pair reads from the Human Genome. The masks used in the main indexes are listed as M, and the masks used in the secondary indexes are listed as S. The key size and key width are indicated next to each mask.

## Table S5

| Mask | Key size (k) and key width (w) | 25bp reads | | | |
| --- | --- | --- | --- | --- | --- |
|  |  | 1 | 2 | 3 | 4 |
| M1 = 111111111111111111 | (k=18, w=18) | M | M | M | M |
| M2 = 11110100110111101010101111 | (k=18, w=26) | M | S |  |  |
| M3 = 11111111111111001111 | (k=18, w=20) | M | S |  |  |
| M4 = 1111011101100101001111111 | (k=18, w=25) | M | S |  |  |
| M5 = 11110111000101010000010101110111 | (k=18, w=32) | M |  |  |  |
| M6 = 1011001101011110100110010010111 | (k=18, w=31) | M |  |  |  |
| M7 = 1110110010100001000101100111001111 | (k=18, w=34) | M |  |  |  |
| M8 = 1111011111111111111 | (k=18, w=19) | M |  |  |  |
| M9 = 11011111100010110111101101 | (k=18, w=26) | M |  |  |  |
| M10 = 111010001110001110100011011111 | (k=18, w=30) | M |  |  |  |

**Index sets used by the four modes of BFAST for 25 base pair ABI SOLiD reads from the human genome.** The masks sets for each of the four BFAST modes: *accurate*, *moderate*.*accuracy*, *moderate speed*, and *fast* are listed under the columns 1, 2, 3, and 4 respectively optimized for 25 base pair reads from the human genome. The masks used in the main indexes are listed as M, and the masks used in the secondary indexes are listed as S. The key size and key width are indicated next to each mask.

## Table S6

| Mask | Key size (k) and key width (w) | 50bp reads | | | |
| --- | --- | --- | --- | --- | --- |
|  |  | 1 | 2 | 3 | 4 |
| M1 = 1111111111111111111111 | (k=22, w=22) | M | M | M | M |
| M2 = 111110100111110011111111111 | (k=22, w=27) | M | S |  |  |
| M3 = 10111111011001100011111000111111 | (k=22, w=32) | M | S |  |  |
| M4 = 1111111100101111000001100011111011 | (k=22, w=34) | M | S |  |  |
| M5 = 111111110001111110011111111 | (k=22, w=27) | M |  |  |  |
| M6 = 11111011010011000011000110011111111 | (k=22, w=35) | M |  |  |  |
| M7 = 1111111111110011101111111 | (k=22, w=25) | M |  |  |  |
| M8 = 111011000011111111001111011111 | (k=22, w=30) | M |  |  |  |
| M9 = 1111111111110011101111111 | (k=22, w=34) | M |  |  |  |
| M10 = 1110110001011010011100101111101111 | (k=22, w=39) | M |  |  |  |

**Index sets used by the four modes of BFAST for 50 base pair ABI SOLiD reads from the human genome.** The masks sets for each of the four BFAST modes: *accurate*, *moderate*.*accuracy*, *moderate speed*, and *fast* are listed under the columns 1, 2, 3, and 4 respectively optimized for 50 base pair reads from the human genome. The masks used in the main indexes are listed as M, and the masks used in the secondary indexes are listed as S. The key size and key width are indicated next to each mask

## Table S7

| **MM** | **0BP** | **1BP(DEL)** | **2BP(1)** | **2BP(2)** | **2BP(3)** | **2BP(4)** | **2BP(5)** | **2BP(6)** | **2BP(7)** | **2BP(8)** | **2BP(9)** | **2BP(10)** |
| --- | --- | --- | --- | --- | --- | --- | --- | --- | --- | --- | --- | --- |
| 0 | 1 | 1 | 1 | 1 | 1 | 1 | 1 | 0.966 | 0.931 | 0.894 | 0.854 | 0.808 |
| 1 | 1 | 0.964 | 0.95 | 0.93 | 0.903 | 0.875 | 0.842 | 0.807 | 0.763 | 0.717 | 0.671 | 0.618 |
| 2 | 1 | 0.851 | 0.825 | 0.792 | 0.757 | 0.717 | 0.674 | 0.633 | 0.587 | 0.538 | 0.486 | 0.435 |
| 3 | 1 | 0.7 | 0.67 | 0.63 | 0.588 | 0.545 | 0.501 | 0.458 | 0.411 | 0.369 | 0.322 | 0.282 |
| 4 | 0.969 | 0.518 | 0.492 | 0.452 | 0.411 | 0.373 | 0.336 | 0.297 | 0.261 | 0.23 | 0.197 | 0.171 |
| 5 | 0.786 | 0.341 | 0.322 | 0.285 | 0.257 | 0.226 | 0.203 | 0.177 | 0.154 | 0.132 | 0.115 | 0.096 |
| 6 | 0.504 | 0.195 | 0.182 | 0.163 | 0.142 | 0.126 | 0.11 | 0.095 | 0.084 | 0.072 | 0.061 | 0.052 |
| 7 | 0.267 | 0.1 | 0.094 | 0.082 | 0.073 | 0.063 | 0.055 | 0.049 | 0.042 | 0.037 | 0.031 | 0.026 |
| 8 | 0.124 | 0.048 | 0.045 | 0.04 | 0.035 | 0.03 | 0.027 | 0.024 | 0.02 | 0.018 | 0.015 | 0.013 |
| 9 | 0.052 | 0.02 | 0.019 | 0.018 | 0.016 | 0.013 | 0.012 | 0.01 | 0.009 | 0.008 | 0.007 | 0.006 |
| 10 | 0.02 | 0.008 | 0.008 | 0.007 | 0.006 | 0.005 | 0.005 | 0.004 | 0.004 | 0.003 | 0.003 | 0.002 |

**The theoretical accuracy statistics for 36 base pair reads for the indexes for the**

***accurate* mode of BFAST shown in Table S3.** The table entries are the fraction of reads for which the correct alignment location will be included in the set of candidate alignment locations (CALs). The ﬁrst column indicates the number of mismatches between the read and the reference, while the subsequence columns indicate the number and type of breakpoints in the read, with 1 breakpoint corresponding to any size deletion, and 2 breakpoints corresponding to an insertion, with the number of inserted bases listed in parentheses. Mismatches are modeled as color errors (denoted CE) for ABI SOLiD data. Accuracy at looking up reads that only have mismatches and no indels is given in the second column, and accuracy against various combined mismatch-indel scenarios is given in the subsequent rows.

## Table S8

| **MM** | **0BP** | **1BP(DEL)** | **2BP(1)** | **2BP(2)** | **2BP(3)** | **2BP(4)** | **2BP(5)** | **2BP(6)** | **2BP(7)** | **2BP(8)** | **2BP(9)** | **2BP(10)** |
| --- | --- | --- | --- | --- | --- | --- | --- | --- | --- | --- | --- | --- |
| 0 | 1 | 1 | 1 | 1 | 1 | 1 | 1 | 0.966 | 0.93 | 0.891 | 0.852 | 0.809 |
| 1 | 1 | 0.895 | 0.875 | 0.851 | 0.824 | 0.789 | 0.755 | 0.72 | 0.675 | 0.638 | 0.597 | 0.555 |
| 2 | 0.875 | 0.665 | 0.644 | 0.614 | 0.586 | 0.553 | 0.52 | 0.49 | 0.458 | 0.422 | 0.392 | 0.359 |
| 3 | 0.634 | 0.443 | 0.426 | 0.4 | 0.378 | 0.357 | 0.334 | 0.312 | 0.287 | 0.266 | 0.243 | 0.218 |
| 4 | 0.409 | 0.274 | 0.259 | 0.246 | 0.233 | 0.216 | 0.203 | 0.189 | 0.17 | 0.158 | 0.142 | 0.131 |
| 5 | 0.246 | 0.157 | 0.151 | 0.144 | 0.132 | 0.125 | 0.116 | 0.108 | 0.099 | 0.09 | 0.081 | 0.072 |
| 6 | 0.14 | 0.088 | 0.083 | 0.078 | 0.075 | 0.068 | 0.064 | 0.058 | 0.054 | 0.047 | 0.043 | 0.039 |
| 7 | 0.074 | 0.046 | 0.044 | 0.041 | 0.038 | 0.036 | 0.033 | 0.03 | 0.027 | 0.025 | 0.023 | 0.02 |
| 8 | 0.038 | 0.024 | 0.022 | 0.02 | 0.019 | 0.018 | 0.016 | 0.015 | 0.014 | 0.012 | 0.011 | 0.01 |
| 9 | 0.018 | 0.01 | 0.01 | 0.009 | 0.009 | 0.009 | 0.007 | 0.007 | 0.007 | 0.006 | 0.005 | 0.004 |
| 10 | 0.008 | 0.005 | 0.005 | 0.004 | 0.004 | 0.004 | 0.003 | 0.003 | 0.003 | 0.003 | 0.002 | 0.002 |

**The theoretical accuracy statistics for 36 base pair reads for the indexes for the *fast***

**mode of BFAST shown in Table S3.** The table format is described in Table S7**.**

## Table S9

| **MM** | **0BP** | **1BP(DEL)** | **2BP(1)** | **2BP(2)** | **2BP(3)** | **2BP(4)** | **2BP(5)** | **2BP(6)** | **2BP(7)** | **2BP(8)** | **2BP(9)** | **2BP(10)** |
| --- | --- | --- | --- | --- | --- | --- | --- | --- | --- | --- | --- | --- |
| 0 | 1 | 1 | 1 | 1 | 1 | 1 | 1 | 1 | 1 | 0.977 | 0.952 | 0.925 |
| 1 | 1 | 0.998 | 0.994 | 0.987 | 0.974 | 0.956 | 0.939 | 0.918 | 0.896 | 0.87 | 0.84 | 0.813 |
| 2 | 1 | 0.941 | 0.923 | 0.905 | 0.881 | 0.858 | 0.834 | 0.808 | 0.779 | 0.752 | 0.72 | 0.687 |
| 3 | 1 | 0.840 | 0.82 | 0.798 | 0.771 | 0.740 | 0.715 | 0.685 | 0.655 | 0.624 | 0.59 | 0.557 |
| 4 | 1 | 0.724 | 0.701 | 0.679 | 0.646 | 0.620 | 0.591 | 0.558 | 0.524 | 0.489 | 0.452 | 0.423 |
| 5 | 0.986 | 0.594 | 0.574 | 0.543 | 0.517 | 0.487 | 0.453 | 0.422 | 0.391 | 0.356 | 0.325 | 0.292 |
| 6 | 0.901 | 0.449 | 0.433 | 0.404 | 0.379 | 0.350 | 0.323 | 0.293 | 0.268 | 0.242 | 0.216 | 0.193 |
| 7 | 0.715 | 0.310 | 0.293 | 0.273 | 0.251 | 0.231 | 0.21 | 0.19 | 0.169 | 0.151 | 0.131 | 0.115 |
| 8 | 0.487 | 0.192 | 0.183 | 0.167 | 0.154 | 0.138 | 0.124 | 0.111 | 0.099 | 0.087 | 0.076 | 0.066 |
| 9 | 0.29 | 0.110 | 0.103 | 0.094 | 0.087 | 0.077 | 0.071 | 0.062 | 0.054 | 0.049 | 0.042 | 0.035 |
| 10 | 0.157 | 0.057 | 0.054 | 0.049 | 0.044 | 0.039 | 0.036 | 0.033 | 0.028 | 0.025 | 0.02 | 0.018 |

**The theoretical accuracy statistics for 50 base pair reads for the indexes for the**

***accurate* mode of BFAST shown in Table S4.** The table format is described in Table S7.

## Table S10

| **MM** | **0BP** | **1BP(DEL)** | **2BP(1)** | **2BP(2)** | **2BP(3)** | **2BP(4)** | **2BP(5)** | **2BP(6)** | **2BP(7)** | **2BP(8)** | **2BP(9)** | **2BP(10)** |
| --- | --- | --- | --- | --- | --- | --- | --- | --- | --- | --- | --- | --- |
| 0 | 1 | 1 | 1 | 1 | 1 | 1 | 1 | 1 | 1 | 0.976 | 0.952 | 0.925 |
| 1 | 1 | 0.901 | 0.888 | 0.873 | 0.857 | 0.835 | 0.813 | 0.79 | 0.763 | 0.736 | 0.708 | 0.681 |
| 2 | 0.887 | 0.682 | 0.668 | 0.649 | 0.625 | 0.601 | 0.578 | 0.556 | 0.536 | 0.511 | 0.489 | 0.467 |
| 3 | 0.664 | 0.466 | 0.451 | 0.439 | 0.417 | 0.404 | 0.384 | 0.368 | 0.352 | 0.335 | 0.317 | 0.299 |
| 4 | 0.444 | 0.299 | 0.287 | 0.274 | 0.266 | 0.253 | 0.241 | 0.234 | 0.218 | 0.206 | 0.194 | 0.184 |
| 5 | 0.278 | 0.181 | 0.176 | 0.169 | 0.16 | 0.153 | 0.144 | 0.14 | 0.13 | 0.125 | 0.116 | 0.11 |
| 6 | 0.168 | 0.106 | 0.103 | 0.098 | 0.094 | 0.087 | 0.085 | 0.081 | 0.075 | 0.071 | 0.068 | 0.063 |
| 7 | 0.094 | 0.059 | 0.057 | 0.054 | 0.052 | 0.051 | 0.047 | 0.046 | 0.042 | 0.04 | 0.037 | 0.035 |
| 8 | 0.052 | 0.032 | 0.031 | 0.03 | 0.028 | 0.027 | 0.026 | 0.024 | 0.022 | 0.021 | 0.02 | 0.019 |
| 9 | 0.027 | 0.016 | 0.016 | 0.016 | 0.015 | 0.014 | 0.013 | 0.013 | 0.012 | 0.011 | 0.011 | 0.01 |
| 10 | 0.014 | 0.008 | 0.009 | 0.008 | 0.007 | 0.007 | 0.006 | 0.006 | 0.006 | 0.006 | 0.005 | 0.005 |

**The theoretical accuracy statistics for 50 base pair reads for the indexes for the**

***fast* mode of BFAST shown in Table S4.** The table format is described in Table S7.

## Table S11

| **CE** | **0BP** | **1BP(DEL)** | **2BP(1)** | **2BP(2)** | **2BP(3)** | **2BP(4)** | **2BP(5)** | **2BP(6)** | **2BP(7)** | **2BP(8)** | **2BP(9)** | **2BP(10)** |
| --- | --- | --- | --- | --- | --- | --- | --- | --- | --- | --- | --- | --- |
| 0 | 1.000 | 0.542 | 0.457 | 0.389 | 0.324 | 0.245 | 0.156 | 0.052 | 0 | 0 | 0 | 0 |
| 1 | 0.960 | 0.311 | 0.250 | 0.187 | 0.141 | 0.079 | 0.049 | 0.016 | 0 | 0 | 0 | 0 |
| 2 | 0.530 | 0.132 | 0.097 | 0.070 | 0.046 | 0.025 | 0.014 | 0.004 | 0 | 0 | 0 | 0 |
| 3 | 0.186 | 0.040 | 0.026 | 0.021 | 0.014 | 0.008 | 0.003 | 0.002 | 0 | 0 | 0 | 0 |
| 4 | 0.044 | 0.008 | 0.006 | 0.004 | 0.003 | 0.002 | 0.001 | 0.001 | 0 | 0 | 0 | 0 |
| 5 | 0.009 | 0.002 | 0.001 | 0.001 | 0 | 0 | 0 | 0 | 0 | 0 | 0 | 0 |
| 6 | 0.001 | 0 | 0 | 0 | 0 | 0 | 0 | 0 | 0 | 0 | 0 | 0 |
| 7 | 0 | 0 | 0 | 0 | 0 | 0 | 0 | 0 | 0 | 0 | 0 | 0 |
| 8 | 0 | 0 | 0 | 0 | 0 | 0 | 0 | 0 | 0 | 0 | 0 | 0 |
| 9 | 0 | 0 | 0 | 0 | 0 | 0 | 0 | 0 | 0 | 0 | 0 | 0 |
| 10 | 0 | 0 | 0 | 0 | 0 | 0 | 0 | 0 | 0 | 0 | 0 | 0 |

**The theoretical accuracy statistics for 25 base pair reads for the indexes for the**

***accurate* mode of BFAST shown in Table S5.** The table format is described in Table S7.

## Table S12

| **CE** | **0BP** | **1BP(DEL)** | **2BP(1)** | **2BP(2)** | **2BP(3)** | **2BP(4)** | **2BP(5)** | **2BP(6)** | **2BP(7)** | **2BP(8)** | **2BP(9)** | **2BP(10)** |
| --- | --- | --- | --- | --- | --- | --- | --- | --- | --- | --- | --- | --- |
| 0 | 1 | 0.538 | 0.455 | 0.389 | 0.321 | 0.239 | 0.149 | 0.051 | 0 | 0 | 0 | 0 |
| 1 | 0.57 | 0.21 | 0.179 | 0.136 | 0.107 | 0.078 | 0.044 | 0.018 | 0 | 0 | 0 | 0 |
| 2 | 0.214 | 0.067 | 0.051 | 0.041 | 0.031 | 0.02 | 0.013 | 0.004 | 0 | 0 | 0 | 0 |
| 3 | 0.06 | 0.018 | 0.013 | 0.013 | 0.01 | 0.006 | 0.004 | 0.001 | 0 | 0 | 0 | 0 |
| 4 | 0.013 | 0.004 | 0.003 | 0.003 | 0.002 | 0.002 | 0 | 0 | 0 | 0 | 0 | 0 |
| 5 | 0.003 | 0.001 | 0.001 | 0.001 | 0 | 0 | 0 | 0 | 0 | 0 | 0 | 0 |
| 6 | 0 | 0 | 0 | 0 | 0 | 0 | 0 | 0 | 0 | 0 | 0 | 0 |
| 7 | 0 | 0 | 0 | 0 | 0 | 0 | 0 | 0 | 0 | 0 | 0 | 0 |
| 8 | 0 | 0 | 0 | 0 | 0 | 0 | 0 | 0 | 0 | 0 | 0 | 0 |
| 9 | 0 | 0 | 0 | 0 | 0 | 0 | 0 | 0 | 0 | 0 | 0 | 0 |
| 10 | 0 | 0 | 0 | 0 | 0 | 0 | 0 | 0 | 0 | 0 | 0 | 0 |

**The theoretical accuracy statistics for 25 base pair reads for the indexes for the**

***fast* mode of BFAST shown in Table S5.** The table format is described in Table S7.

## Table S13

| **CE** | **0BP** | **1BP(DEL)** | **2BP(1)** | **2BP(2)** | **2BP(3)** | **2BP(4)** | **2BP(5)** | **2BP(6)** | **2BP(7)** | **2BP(8)** | **2BP(9)** | **2BP(10)** |
| --- | --- | --- | --- | --- | --- | --- | --- | --- | --- | --- | --- | --- |
| 0 | 1 | 1 | 1 | 1 | 1 | 1 | 1 | 0.977 | 0.953 | 0.929 | 0.901 | 0.87 |
| 1 | 1 | 0.999 | 0.984 | 0.971 | 0.957 | 0.934 | 0.914 | 0.891 | 0.859 | 0.838 | 0.802 | 0.777 |
| 2 | 1 | 0.951 | 0.909 | 0.89 | 0.87 | 0.838 | 0.823 | 0.792 | 0.764 | 0.733 | 0.704 | 0.663 |
| 3 | 1 | 0.85 | 0.817 | 0.785 | 0.757 | 0.735 | 0.716 | 0.69 | 0.646 | 0.613 | 0.583 | 0.551 |
| 4 | 0.999 | 0.745 | 0.703 | 0.674 | 0.655 | 0.617 | 0.599 | 0.571 | 0.528 | 0.494 | 0.459 | 0.42 |
| 5 | 0.975 | 0.612 | 0.568 | 0.538 | 0.513 | 0.493 | 0.447 | 0.425 | 0.392 | 0.368 | 0.331 | 0.308 |
| 6 | 0.867 | 0.46 | 0.425 | 0.401 | 0.374 | 0.356 | 0.317 | 0.297 | 0.28 | 0.252 | 0.226 | 0.201 |
| 7 | 0.683 | 0.318 | 0.29 | 0.272 | 0.247 | 0.23 | 0.217 | 0.189 | 0.179 | 0.156 | 0.15 | 0.128 |
| 8 | 0.462 | 0.199 | 0.178 | 0.167 | 0.155 | 0.143 | 0.129 | 0.116 | 0.11 | 0.091 | 0.083 | 0.072 |
| 9 | 0.282 | 0.113 | 0.107 | 0.099 | 0.084 | 0.081 | 0.08 | 0.069 | 0.057 | 0.053 | 0.044 | 0.037 |
| 10 | 0.163 | 0.063 | 0.064 | 0.056 | 0.049 | 0.042 | 0.041 | 0.035 | 0.03 | 0.029 | 0.027 | 0.022 |

**The theoretical accuracy statistics for 50 base pair reads for the indexes for the**

***accurate* mode of BFAST shown in Table S6.** The table format is described in Table S7**.**

## Table S14

| **CE** | **0BP** | **1BP(DEL)** | **2BP(1)** | **2BP(2)** | **2BP(3)** | **2BP(4)** | **2BP(5)** | **2BP(6)** | **2BP(7)** | **2BP(8)** | **2BP(9)** | **2BP(10)** |
| --- | --- | --- | --- | --- | --- | --- | --- | --- | --- | --- | --- | --- |
| 0 | 1 | 1 | 1 | 1 | 1 | 1 | 1 | 0.975 | 0.954 | 0.925 | 0.904 | 0.875 |
| 1 | 1 | 0.889 | 0.863 | 0.842 | 0.82 | 0.8 | 0.778 | 0.741 | 0.722 | 0.692 | 0.663 | 0.644 |
| 2 | 0.889 | 0.671 | 0.627 | 0.611 | 0.579 | 0.566 | 0.54 | 0.533 | 0.503 | 0.474 | 0.457 | 0.435 |
| 3 | 0.666 | 0.449 | 0.431 | 0.418 | 0.398 | 0.375 | 0.362 | 0.344 | 0.326 | 0.312 | 0.296 | 0.279 |
| 4 | 0.448 | 0.295 | 0.264 | 0.257 | 0.254 | 0.229 | 0.227 | 0.21 | 0.205 | 0.193 | 0.184 | 0.165 |
| 5 | 0.286 | 0.178 | 0.166 | 0.164 | 0.155 | 0.146 | 0.138 | 0.131 | 0.12 | 0.112 | 0.112 | 0.104 |
| 6 | 0.17 | 0.099 | 0.095 | 0.091 | 0.086 | 0.084 | 0.074 | 0.082 | 0.068 | 0.068 | 0.062 | 0.06 |
| 7 | 0.097 | 0.06 | 0.057 | 0.055 | 0.05 | 0.048 | 0.044 | 0.044 | 0.041 | 0.038 | 0.033 | 0.032 |
| 8 | 0.05 | 0.034 | 0.027 | 0.028 | 0.025 | 0.025 | 0.022 | 0.022 | 0.024 | 0.017 | 0.018 | 0.017 |
| 9 | 0.028 | 0.019 | 0.017 | 0.013 | 0.013 | 0.013 | 0.015 | 0.011 | 0.012 | 0.01 | 0.01 | 0.009 |
| 10 | 0.014 | 0.009 | 0.007 | 0.007 | 0.007 | 0.006 | 0.006 | 0.007 | 0.006 | 0.005 | 0.005 | 0.005 |

**The theoretical accuracy statistics for 50 base pair reads for the indexes for the**

***fast* mode of BFAST shown in Table S6.** The table format is described in Table S7.

## Table S15

| **Dataset** | **Read length** | **Original number of reads** | **Number of computer cpus required to map 1 billion reads for a given BFAST mode in one day** | | | |
| --- | --- | --- | --- | --- | --- | --- |
|  |  |  | ***accurate*** | ***moderate.accuracy*** | ***moderate.speed*** | ***fast*** |
| Illumina | 36 | 10.9M | 47 | 12 | 9 | 1 |
| Illumina | 55 | 3.5M | 156 | 34 | 23 | 3 |
| ABI SOLiD | 25 | 1M | 111 | 54 | 46 | 16 |
| ABI SOLiD | 50 | 1M | 496 | 124 | 90 | 20 |

**Assessing our goal of mapping 1 billion reads in one day.** We assess to what extent we have met the billion reads per day goal by extrapolating the performance from the real-world datasets. We give the number of computer cpus required to map one billion reads per day for each of the four BFAST modes presented. This analysis ignores cpus that contain multiple cores, which could dramatically reduce the number of cpus required. See the Supplementary Methods for the description of each dataset.

# References

1. Smith TF, Waterman MS (1981) Identification of common molecular subsequences. J Mol Biol 147: 195-197.

2. Korf RE, Zhang W, Thayer G, Hohwald H (2005) Frontier search. J ACM 52: 715-748.

3. Navarro G (2001) A guided tour to approximate string matching. ACM Comput Surv 33: 31-88.

4. Ukkonen E (1993) Approximate String-Matching over Suffix Trees. Proceedings of the 4th Annual Symposium on Combinatorial Pattern Matching: Springer-Verlag.

5. Trinh NDH, Wing-Kai H, Tak-Wah L, Wing-Kin S (2006) Approximate string matching using compressed suffix arrays. Theor Comput Sci 352: 240-249.

6. Altschul SF, Madden TL, Schaffer AA, Zhang J, Zhang Z, et al. (1997) Gapped BLAST and PSI-BLAST: a new generation of protein database search programs. Nucleic Acids Res 25: 3389-3402.

7. Cox A (Unpublished 2007) ELAND: Efficient Local Alignment of Nucleotide Data.

8. Kent WJ (2002) BLAT--the BLAST-like alignment tool. Genome Res 12: 656-664.

9. Li R, Li Y, Kristiansen K, Wang J (2008) SOAP: short oligonucleotide alignment program. Bioinformatics 24: 713-714.

10. Ning Z, Cox AJ, Mullikin JC (2001) SSAHA: a fast search method for large DNA databases. Genome Res 11: 1725-1729.

11. Rumble SM, Lacroute P, Dalca AV, Fiume M, Sidow A, et al. (2008) SHRiMP - Short Reading Mapping Package.

12. Li H, Ruan J, Durbin R (2008) Mapping short DNA sequencing reads and calling variants using mapping quality scores. Genome Res 18: 1851-1858.

13. Sun Y, Buhler J (2005) Designing multiple simultaneous seeds for DNA similarity search. J Comput Biol 12: 847-861.

14. Ilie L, Ilie S (2007) Multiple spaced seeds for homology search. Bioinformatics 23: 2969-2977.

15. Ma B, Tromp J, Li M (2002) PatternHunter: faster and more sensitive homology search. Bioinformatics 18: 440-445.

16. Needleman SB, Wunsch CD (1970) A general method applicable to the search for similarities in the amino acid sequence of two proteins. J Mol Biol 48: 443-453.

17. Homer N, Merriman B, Nelson SF (Unpublished) Local alignment of two-base encoded DNA sequence.
